# Supplementary material for: Investigating the relationship of COVID-19 related stress and media consumption with schizotypy, depression, and anxiety in cross-sectional surveys repeated throughout the pandemic in Germany and the UK
Source: eLife. 2022 Jul 4;11:e75893. doi: 10.7554/eLife.75893 (PMC9252577; doi:10.7554/eLife.75893)
Supplement: Supplementary file 1. — (a) Overview of the model fit indices for predictor models separated by time point. (b) Results of Generalised Estimate Equation models for the three outcome variables with survey time point as predictor. (c) Pearson’s correlation coefficient between SPQ sumscore, anxiety score (SCL-27) and depression score (SCL-27) at all four survey time points. (d) Overview of the model fit indices separated by exogeneous latent variable and survey excluding 27 subjects, who completed all four time points. (e) Complete outcome of structural equation with COVID-19 related life concerns from first survey timepoint. (f) Complete outcome of structural equation with COVID-19 related life concerns from second survey time point. (g) Complete outcome of structural equation with COVID-19 related life concerns from third survey time point. (h) Complete outcome of structural equation with COVID-19 related life concerns from fourth survey time point. (i) Complete outcome of structural equation with Social adversity as predictor from the first survey time point. (j) Complete outcome of structural equation with Social adversity as predictor from second survey time point. (k) Complete outcome of structural equation with Social adversity as predictor from third survey time point. (l) Complete outcome of structural equation with Social adversity as predictor from fourth survey time point. (m) Model fit for model with reduced complexity, one predictor, one outcome, five mediators, example for time point 1. (n) Model comparison for original and alternative model. (o) Overview of the model fit indices separated by exogeneous latent variable and time point. [file elife-75893-supp1.docx]

SUPPLEMENTARY MATERIAL

Investigating the relationship of COVID-19 related stress and media consumption with schizotypy, depression and anxiety in cross-sectional surveys repeated throughout the pandemic in Germany and the UK

**Sarah Daimer^a^, Lorenz Mihatsch^b,c^, Sharon Neufeld^d^, Graham K. Murray^d,e^, Franziska Knolle^a,d*^**

^a^ Department of Diagnostic and Interventional Neuroradiology, School of Medicine, Technical University of Munich, Munich, Germany.

^b^ Department of Anesthesiology and Intensive Care Medicine, Ludwig-Maximilians-Universität München, Munich, Germany
^c^ Institute for Medical Information Processing, Biometry and Epidemiology, Ludwig-Maximilians-Universität München, Munich, Germany.

^d^ Department of Psychiatry, University of Cambridge, Cambridge UK.

^e^ Cambridgeshire and Peterborough NHS Foundation Trust, Cambridge, United Kingdom.

*corresponding author: franziska.knolle@tum.de

Table of Contents

[1 Model fit predictor measurement models 3](#_Toc102385120)

[2 Generalised Estimate Equation models for SPQ, Anxiety and Depression scores 3](#_Toc102385121)

[3 Pearson’s correlation of anxiety and SPQ scores and depression and SPQ scores 3](#_Toc102385122)

[4 Model fit indices without 27 participants who participated four times 4](#_Toc102385123)

[5 Complete outcome of structural equation models 4](#_Toc102385124)

[5.1 COVID-19 related life concerns’ model – first to forth timepoint 4](#_Toc102385125)

[5.1.1 First Timepoint 4](#_Toc102385126)

[5.1.2 Second Timepoint 9](#_Toc102385127)

[5.1.3 Third timepoint 14](#_Toc102385128)

[5.1.4 Fourth timepoint 19](#_Toc102385129)

[5.2 ‘Social adversity’ Model – first to forth timepoint 24](#_Toc102385130)

[5.2.1 Social adversity Model – first timepoint 24](#_Toc102385131)

[5.2.2 Social adversity Model – second timepoint 29](#_Toc102385132)

[5.2.3 Social adversity Model – third timepoint 34](#_Toc102385133)

[5.2.4 Social adversity Model – fourth timepoint 39](#_Toc102385134)

[6 Model with reduced in complexity, with one predictor and one outcome without control variables 44](#_Toc102385135)

[7 Alternative models 45](#_Toc102385136)

[7.1 COVID-19 related life concerns’ model – alternative models 45](#_Toc102385137)

[7.2 ‘Social adversity’ Model – alternative models 45](#_Toc102385138)

[8 Exploratory model COVID-stress -> Anxiety/Depression -> SPQ 46](#_Toc102385139)

# Model fit predictor measurement models

| **Suppl. file 1a. Overview of the model fit indices for predictor models separated by timepoint** |
| --- |

|  |  |  |  | exact modelfit |  | relativ modelfit | | absolute modelfit | |
| --- | --- | --- | --- | --- | --- | --- | --- | --- | --- |
|  |  | Teststatistic | DF | *X^2^* |  | CFI |  | | RMSEA |
| Predictor | Timepoint |  |  |  |  |  |  | |  |
| COVID-19 related life concerns | 1 | 163.73 | 3 | .000 |  | 1.0 |  | | 0.0 |
|  | 2 | 201.93 | 3 | .000 |  | 1.0 |  | | 0.0 |
|  | 3 | 326.03 | 3 | .000 |  | 1.0 |  | | 0.0 |
|  | 4 | 242.49 | 3 | .000 |  | 1.0 |  | | 0.0 |
| Social adversity | 1 | 269.48 | 3 | .000 |  | 1.0 |  | | 0.0 |
|  | 2 | 258.30 | 3 | .000 |  | 1.0 |  | | 0.0 |
|  | 3 | 364.40 | 3 | .000 |  | 1.0 |  | | 0.0 |
|  | 4 | 335.31 | 3 | .000 |  | 1.0 |  | | 0.0 |
| DF: degree of freedom, *X^2^*: Chi squared test, CFI: comparative fit index, TLI: Tucker-Lewis index, RMSEA: root mean square error of approximation | | | | | | | | | |

# Generalised Estimate Equation models for SPQ, Anxiety and Depression scores

| **Suppl. file 1b. Results of Generalised Estimate Equation models for the three outcome variables with survey timepoint as predictor** | | | | | | | | | | |
| --- | --- | --- | --- | --- | --- | --- | --- | --- | --- | --- |
|  | | SPQ | | |  | Anxiety | |  | Depression | |
|  | | Coefficient | p-value | |  | Coefficient | p-value |  | Coefficient | p-value |
| Intercept | | 12.70 | .000 | |  | 0.56 | .000 |  | 0.86 | .000 |
| Sept./ Oct. 20 | | 1.28 | .051 | |  | -0.15 | .000 |  | -0.17 | .000 |
| Jan. /Feb. 21 | | 0.68 | .288 | |  | -0.15 | .000 |  | -0.04 | .343 |
| May 21 | | 0.74 | .261 | |  | -0.12 | .000 |  | -0.03 | .507 |
|  |  | | |  | | | | | | |

# Pearson’s correlation of anxiety and SPQ scores and depression and SPQ scores

| **Suppl. file 1c. Pearson’s correlation coefficient between SPQ sumscore, anxiety score (SCL-27) and depression score (SCL-27) at all four survey timepoints** | | | |
| --- | --- | --- | --- |
| Survey | SPQ &  Anxiety | SPQ &  Depression | Anxiety &  Depression |
| 1 - April/May 20 | 0.57 *** | 0.53 *** | 0.68 *** |
| 2 - Sept./ Oct. 20 | 0.68 *** | 0.58 *** | 0.73 *** |
| 3 - Jan. /Feb. 21 | 0.67 *** | 0.56 *** | 0.73 *** |
| 4 - May 21 | 0.66 *** | 0.56 *** | 0.71 *** |
| *** = p <.001 | | |  |

# Model fit indices without 27 participants who participated four times

| **Suppl. file 1d. Overview of the model fit indices separated by exogeneous latent variable and survey excluding 27 subjects, who completed all four time points.** | | | | | | | | | | | |
| --- | --- | --- | --- | --- | --- | --- | --- | --- | --- | --- | --- |
|  |  |  |  | exact model fit |  | relative model fit | |  | | absolute model fit |  |
|  |  | Test statistic | df | *X^2^* |  | CFI |  | | RMSEA | | |
| Predictor | Survey |  |  |  |  |  |  | |  | | |
| COVID-19 related  life  concerns | 1 - April/May 20 | 473.72 | 135 | .000 |  | 0.834 |  | | 0.073 | | |
|  | 2 - Sept./ Oct. 20 | 432.84 | 135 | .000 |  | 0.857 |  | | 0.074 | | |
|  | 3 - Jan. /Feb. 21 | 411.69 | 135 | .000 |  | 0.885 |  | | 0.066 | | |
|  | 4 - May 21 | 392.08 | 135 | .000 |  | 0.868 |  | | 0.067 | | |
| Social adversity | 1 - April/May 20 | 487.92 | 135 | .000 |  | 0.851 |  | | 0.075 | | |
|  | 2 - Sept./ Oct. 20 | 466.64 | 135 | .000 |  | 0.856 |  | | 0.078 | | |
|  | 3 - Jan. /Feb. 21 | 479.34 | 135 | .000 |  | 0.868 |  | | 0.074 | | |
|  | 4 - May 21 | 420.65 | 135 | .000 |  | 0.869 |  | | 0.071 | | |
| df: degree of freedom, *X^2^*: Chi squared test, CFI: comparative fit index, TLI: Tucker-Lewis index, RMSEA: root mean square error of approximation | | | | | | | | | | | |

# Complete outcome of structural equation models

## COVID-19 related life concerns’ model – first to forth timepoint

### First Timepoint

| **Suppl. file 1e. Complete outcome of structural equation with COVID-19 related life concerns from first survey timepoint.** | | | | | | |
| --- | --- | --- | --- | --- | --- | --- |
| **Estimator** | **ML** |  |  |  |  |  |
| Optimization method | NLMINB |  |  |  |  |  |
| Number of free parameters | 108 |  |  |  |  |  |
| Number of observations | 480 |  |  |  |  |  |
| **Model Test User Model:** |  |  |  |  |  |  |
|  |  |  |  |  |  |  |
| Test statistic | 480.156 |  |  |  |  |  |
| Degrees of freedom | 135 |  |  |  |  |  |
| P-value (Chi-square) | 0.000 |  |  |  |  |  |
| **Model Test Baseline Model:** |  |  |  |  |  |  |
| Test statistic | 2326.466 |  |  |  |  |  |
| Degrees of freedom | 225 |  |  |  |  |  |
| P-value | 0.000 |  |  |  |  |  |
| **User Model versus Baseline Model:** |  |  |  |  |  |  |
| Comparative Fit Index (CFI) | 0.836 |  |  |  |  |  |
| Tucker-Lewis Index (TLI) | 0.726 |  |  |  |  |  |
| **Loglikelihood and Information Criteria:** |  |  |  |  |  |  |
| Loglikelihood user model (H0) | -10237.799 |  |  |  |  |  |
| Loglikelihood unrestricted model (H1) | -9997.722 |  |  |  |  |  |
| Akaike (AIC) | 20691.599 |  |  |  |  |  |
| Bayesian (BIC) | 21142.368 |  |  |  |  |  |
| Sample-size adjusted Bayesian (BIC) | 20799.587 |  |  |  |  |  |
| **Root Mean Square Error of Approximation:** |  |  |  |  |  |  |
| RMSEA | 0.073 |  |  |  |  |  |
| 90 Percent confidence interval - lower | 0.066 |  |  |  |  |  |
| 90 Percent confidence interval - upper | 0.080 |  |  |  |  |  |
| P-value RMSEA <= 0.05 | 0.000 |  |  |  |  |  |
| **Standardized Root Mean Square Residual:** |  |  |  |  |  |  |
| SRMR | 0.067 |  |  |  |  |  |
| **Parameter Estimates:** |  |  |  |  |  |  |
| Standard errors | Bootstrap |  |  |  |  |  |
| Number of requested bootstrap draws | 1000 |  |  |  |  |  |
| Number of successful bootstrap draws | 1000 |  |  |  |  |  |
| **Latent Variables:** |  |  |  |  |  |  |
|  | Estimate | Std.Err | z-value | P(>\|z\|) | Std.lv | Std.all |
| Living Conditions =~ |  |  |  |  |  |  |
| Number_rooms | 1.000 |  |  |  | 2.201 | 0.789 |
| Number people per household | 0.232 | 0.023 | 10.299 | 0.000 | 0.510 | 0.642 |
| Living Area | 0.324 | 0.030 | 10.924 | 0.000 | 0.713 | 0.528 |
| Garden_yard | 0.132 | 0.014 | 9.264 | 0.000 | 0.290 | 0.630 |
| Health before COVID19 =~ | |  |  |  |  |  |
| Regular treatment physical illness | 1.000 |  |  |  | 0.163 | 0.477 |
| Mental health status before COVID19 | 5.523 | 0.981 | 5.628 | 0.000 | 0.898 | 0.815 |
| Physical health status before COVID19 | 3.489 | 0.628 | 5.554 | 0.000 | 0.567 | 0.574 |
| COVID19 related life concerns =~ | |  |  |  |  |  |
| Financial impact due to COVID19 | 0.593 | 0.124 | 4.798 | 0.000 | 0.593 | 0.498 |
| Concerns about life stability | 1.005 | 0.199 | 5.052 | 0.000 | 1.005 | 0.786 |
| Restrictions perceived as stressful | 0.557 | 0.159 | 3.512 | 0.000 | 0.557 | 0.467 |
|  |  |  |  |  |  |  |
| **Regressions:** |  |  |  |  |  |  |
|  | Estimate | Std.Err | z-value | P(>\|z\|) | Std.lv | Std.all |
| **depression ~** | |  |  |  |  |  |
| Health before COVID19 | 2.306 | 0.348 | 6.626 | 0.000 | 0.375 | 0.533 |
| Country (residence) | -0.284 | 0.067 | -4.210 | 0.000 | -0.284 | -0.182 |
| Age | -0.057 | 0.030 | -1.854 | 0.064 | -0.057 | -0.080 |
| Gender | 0.061 | 0.060 | 1.015 | 0.310 | 0.061 | 0.038 |
| Highest education | -0.031 | 0.022 | -1.447 | 0.148 | -0.031 | -0.054 |
| Living Conditions | 0.009 | 0.018 | 0.483 | 0.629 | 0.019 | 0.027 |
| **anxiety ~** |  |  |  |  |  |  |
| Health before COVID19 | 1.632 | 0.294 | 5.553 | 0.000 | 0.265 | 0.458 |
| Country (residence) | -0.240 | 0.061 | -3.969 | 0.000 | -0.240 | -0.187 |
| Age | -0.085 | 0.024 | -3.542 | 0.000 | -0.085 | -0.146 |
| Gender | 0.032 | 0.049 | 0.656 | 0.512 | 0.032 | 0.025 |
| Highest education | 0.006 | 0.019 | 0.290 | 0.772 | 0.006 | 0.011 |
| Living Conditions | 0.006 | 0.015 | 0.376 | 0.707 | 0.012 | 0.022 |
| **SPQ_total ~** | |  |  |  |  |  |
| Health before COVID19 | 2.671 | 0.488 | 5.469 | 0.000 | 0.434 | 0.447 |
| Country (residence) | 0.028 | 0.112 | 0.247 | 0.805 | 0.028 | 0.013 |
| Age | -0.087 | 0.048 | -1.813 | 0.070 | -0.087 | -0.089 |
| Gender | -0.141 | 0.088 | -1.604 | 0.109 | -0.141 | -0.064 |
| Highest education | -0.063 | 0.040 | -1.586 | 0.113 | -0.063 | -0.078 |
| Living Conditions | -0.020 | 0.024 | -0.857 | 0.392 | -0.045 | -0.046 |
| **Media ~** | |  |  |  |  |  |
| Health before COVID19 | 0.105 | 0.181 | 0.581 | 0.561 | 0.017 | 0.037 |
| Country (residence) | -0.174 | 0.050 | -3.492 | 0.000 | -0.174 | -0.169 |
| Age | -0.070 | 0.021 | -3.309 | 0.001 | -0.070 | -0.151 |
| Gender | -0.009 | 0.047 | -0.193 | 0.847 | -0.009 | -0.009 |
| Highest education | -0.046 | 0.017 | -2.642 | 0.008 | -0.046 | -0.119 |
| Living Conditions | -0.018 | 0.013 | -1.397 | 0.162 | -0.039 | -0.084 |
| **Drugs ~** | |  |  |  |  |  |
| Health before COVID19 | 0.556 | 0.147 | 3.779 | 0.000 | 0.090 | 0.272 |
| Country (residence) | 0.011 | 0.036 | 0.300 | 0.764 | 0.011 | 0.015 |
| Age | 0.025 | 0.016 | 1.562 | 0.118 | 0.025 | 0.075 |
| Gender | -0.007 | 0.035 | -0.209 | 0.835 | -0.007 | -0.010 |
| Highest education | -0.005 | 0.012 | -0.371 | 0.711 | -0.005 | -0.016 |
| Living Conditions | -0.004 | 0.008 | -0.436 | 0.663 | -0.008 | -0.024 |
| **Alcohol ~** | |  |  |  |  |  |
| Health before COVID19 | -1.439 | 0.880 | -1.635 | 0.102 | -0.234 | -0.104 |
| Country (residence) | -0.504 | 0.232 | -2.169 | 0.030 | -0.504 | -0.101 |
| Age | 0.456 | 0.102 | 4.466 | 0.000 | 0.456 | 0.202 |
| Gender | -0.490 | 0.228 | -2.144 | 0.032 | -0.490 | -0.096 |
| Highest education | 0.063 | 0.092 | 0.686 | 0.493 | 0.063 | 0.034 |
| Living Conditions | 0.051 | 0.059 | 0.860 | 0.390 | 0.112 | 0.050 |
| **Exercise ~** | |  |  |  |  |  |
| Health before COVID19 | -1.090 | 0.523 | -2.084 | 0.037 | -0.177 | -0.142 |
| Country (residence) | -0.508 | 0.138 | -3.667 | 0.000 | -0.508 | -0.184 |
| Age | 0.174 | 0.060 | 2.895 | 0.004 | 0.174 | 0.139 |
| Gender | -0.047 | 0.127 | -0.369 | 0.712 | -0.047 | -0.017 |
| Highest education | 0.036 | 0.045 | 0.812 | 0.417 | 0.036 | 0.035 |
| Living Conditions | -0.013 | 0.032 | -0.411 | 0.681 | -0.029 | -0.023 |
| **Sleep during week ~** | |  |  |  |  |  |
| Health before COVID19 | -0.095 | 0.256 | -0.370 | 0.711 | -0.015 | -0.024 |
| Country (residence) | -0.087 | 0.067 | -1.301 | 0.193 | -0.087 | -0.062 |
| Age | -0.075 | 0.029 | -2.575 | 0.010 | -0.075 | -0.118 |
| Gender | 0.102 | 0.067 | 1.526 | 0.127 | 0.102 | 0.071 |
| Highest education | -0.044 | 0.024 | -1.799 | 0.072 | -0.044 | -0.083 |
| Living Conditions | -0.019 | 0.017 | -1.118 | 0.264 | -0.042 | -0.066 |
| **healthBeforeCo19 ~** | |  |  |  |  |  |
| Country (residence) | -0.060 | 0.021 | -2.812 | 0.005 | -0.370 | -0.167 |
| Age | -0.025 | 0.010 | -2.589 | 0.010 | -0.154 | -0.153 |
| Gender | 0.045 | 0.020 | 2.281 | 0.023 | 0.278 | 0.123 |
| Highest education | -0.020 | 0.008 | -2.608 | 0.009 | -0.124 | -0.149 |
| Living Conditions | -0.008 | 0.006 | -1.424 | 0.154 | -0.107 | -0.107 |
| **depression ~** | |  |  |  |  |  |
| COVID19 related life concerns (c1) | 0.217 | 0.076 | 2.851 | 0.004 | 0.217 | 0.309 |
| **anxiety ~** |  |  |  |  |  |  |
| COVID19 related life concerns (c2) | 0.069 | 0.049 | 1.405 | 0.160 | 0.069 | 0.119 |
| **SPQ_total ~** | |  |  |  |  |  |
| COVID19 related life concerns (c3) | -0.004 | 0.065 | -0.063 | 0.950 | -0.004 | -0.004 |
| **Media ~** | |  |  |  |  |  |
| COVID19 related life concerns (a1) | 0.048 | 0.027 | 1.762 | 0.078 | 0.048 | 0.103 |
| **Drugs ~** | |  |  |  |  |  |
| COVID19 related life concerns (a2) | 0.030 | 0.027 | 1.112 | 0.266 | 0.030 | 0.089 |
| **Alcohol ~** | |  |  |  |  |  |
| COVID19 related life concerns (a3) | 0.199 | 0.180 | 1.102 | 0.271 | 0.199 | 0.088 |
| **Exercise ~** | |  |  |  |  |  |
| COVID19 related life concerns (a4) | -0.079 | 0.082 | -0.955 | 0.340 | -0.079 | -0.063 |
| **Sleep during week ~** | |  |  |  |  |  |
| COVID19 related life concerns (a5) | -0.080 | 0.050 | -1.600 | 0.110 | -0.080 | -0.125 |
| **depression ~** | |  |  |  |  |  |
| Media (b1) | 0.084 | 0.061 | 1.372 | 0.170 | 0.084 | 0.056 |
| Drugs (b2) | 0.076 | 0.098 | 0.775 | 0.439 | 0.076 | 0.036 |
| Alcohol (b3) | 0.036 | 0.014 | 2.588 | 0.010 | 0.036 | 0.114 |
| Exercise (b4) | -0.008 | 0.022 | -0.343 | 0.731 | -0.008 | -0.014 |
| Sleep during week(b5) | -0.152 | 0.054 | -2.797 | 0.005 | -0.152 | -0.137 |
| **anxiety ~** |  |  |  |  |  |  |
| Media (b6) | 0.095 | 0.057 | 1.674 | 0.094 | 0.095 | 0.076 |
| Drug (b7) | 0.052 | 0.097 | 0.538 | 0.591 | 0.052 | 0.030 |
| Alcohol (b8) | 0.011 | 0.011 | 0.948 | 0.343 | 0.011 | 0.041 |
| Exercise (b9) | 0.011 | 0.021 | 0.514 | 0.607 | 0.011 | 0.023 |
| Sleep during week (b10) | -0.094 | 0.046 | -2.023 | 0.043 | -0.094 | -0.103 |
| **SPQ_total ~** | |  |  |  |  |  |
| Media (b11) | 0.231 | 0.100 | 2.323 | 0.020 | 0.231 | 0.111 |
| Drug (b12) | 0.147 | 0.148 | 0.998 | 0.318 | 0.147 | 0.050 |
| Alcohol (b13) | 0.010 | 0.021 | 0.489 | 0.625 | 0.010 | 0.024 |
| Exercise (b14) | -0.041 | 0.033 | -1.233 | 0.218 | -0.041 | -0.053 |
| Sleep during week (b15) | -0.118 | 0.066 | -1.791 | 0.073 | -0.118 | -0.077 |
|  |  |  |  |  |  |  |
| **Covariances:** |  |  |  |  |  |  |
|  | Estimate | Std.Err | z-value | P(>\|z\|) | Std.lv | Std.all |
| .anxiety ~~ |  |  |  |  |  |  |
| .SPQ_total | 0.135 | 0.025 | 5.446 | 0.000 | 0.135 | 0.363 |
| .depression ~~ |  |  |  |  |  |  |
| .SPQ_total | 0.058 | 0.026 | 2.222 | 0.026 | 0.058 | 0.151 |
| .anxiety | 0.099 | 0.019 | 5.086 | 0.000 | 0.099 | 0.458 |
| Living Conditions ~~ |  |  |  |  |  |  |
| COVID19 related life concerns | -0.443 | 0.150 | -2.958 | 0.003 | -0.201 | -0.201 |
|  |  |  |  |  |  |  |
| **Variances:** |  |  |  |  |  |  |
|  | Estimate | Std.Err | z-value | P(>\|z\|) | Std.lv | Std.all |
| COVID19 related life concerns | 1.000 |  |  |  | 1.000 | 1.000 |
| .Number rooms | 2.931 | 0.457 | 6.410 | 0.000 | 2.931 | 0.377 |
| .Number people per household | 0.371 | 0.028 | 13.260 | 0.000 | 0.371 | 0.588 |
| .Living Area | 1.318 | 0.098 | 13.503 | 0.000 | 1.318 | 0.722 |
| .Garden_yard | 0.128 | 0.010 | 13.073 | 0.000 | 0.128 | 0.603 |
| .Regular treatment physical illness | 0.089 | 0.009 | 10.132 | 0.000 | 0.089 | 0.772 |
| .Mental health status before COVID19 | 0.409 | 0.098 | 4.164 | 0.000 | 0.409 | 0.336 |
| .Physical health status before COVID19 | 0.655 | 0.050 | 13.064 | 0.000 | 0.655 | 0.671 |
| .Financial Impact due to COVID19 | 1.064 | 0.111 | 9.620 | 0.000 | 1.064 | 0.752 |
| .Concerns about life stability | 0.624 | 0.387 | 1.613 | 0.107 | 0.624 | 0.381 |
| .Restrictions perceived as stressful | 1.114 | 0.195 | 5.717 | 0.000 | 1.114 | 0.782 |
| .depression | 0.224 | 0.033 | 6.752 | 0.000 | 0.224 | 0.453 |
| .anxiety | 0.207 | 0.022 | 9.239 | 0.000 | 0.207 | 0.618 |
| .SPQ_total | 0.664 | 0.061 | 10.812 | 0.000 | 0.664 | 0.705 |
| .Media | 0.193 | 0.009 | 21.552 | 0.000 | 0.193 | 0.896 |
| .Drug | 0.101 | 0.010 | 9.649 | 0.000 | 0.101 | 0.917 |
| .Alcohol | 4.606 | 0.199 | 23.100 | 0.000 | 4.606 | 0.913 |
| .Exercise | 1.438 | 0.076 | 18.908 | 0.000 | 1.438 | 0.929 |
| .Sleep during week | 0.383 | 0.027 | 14.418 | 0.000 | 0.383 | 0.951 |
| Living Conditions | 4.846 | 0.569 | 8.518 | 0.000 | 1.000 | 1.000 |
| .health before COVID19 | 0.023 | 0.006 | 3.620 | 0.000 | 0.886 | 0.886 |
|  |  |  |  |  |  |  |
| **Defined Parameters:** |  |  |  |  |  |  |
|  | Estimate | Std.Err | z-value | P(>\|z\|) | Std.lv | Std.all |
| indirect1 | 0.004 | 0.004 | 1.075 | 0.282 | 0.004 | 0.006 |
| indirect2 | 0.002 | 0.004 | 0.520 | 0.603 | 0.002 | 0.003 |
| indirect3 | 0.007 | 0.006 | 1.250 | 0.211 | 0.007 | 0.010 |
| indirect4 | 0.001 | 0.002 | 0.256 | 0.798 | 0.001 | 0.001 |
| indirect5 | 0.012 | 0.007 | 1.721 | 0.085 | 0.012 | 0.017 |
| indirect6 | 0.005 | 0.004 | 1.174 | 0.240 | 0.005 | 0.008 |
| indirect7 | 0.002 | 0.004 | 0.351 | 0.726 | 0.002 | 0.003 |
| indirect8 | 0.002 | 0.003 | 0.620 | 0.535 | 0.002 | 0.004 |
| indirect9 | -0.001 | 0.002 | -0.390 | 0.696 | -0.001 | -0.001 |
| indirect10 | 0.007 | 0.006 | 1.293 | 0.196 | 0.007 | 0.013 |
| indirect11 | 0.011 | 0.008 | 1.337 | 0.181 | 0.011 | 0.011 |
| indirect12 | 0.004 | 0.008 | 0.581 | 0.561 | 0.004 | 0.005 |
| indirect13 | 0.002 | 0.007 | 0.304 | 0.761 | 0.002 | 0.002 |
| indirect14 | 0.003 | 0.005 | 0.666 | 0.506 | 0.003 | 0.003 |
| indirect15 | 0.009 | 0.009 | 1.021 | 0.307 | 0.009 | 0.010 |
| total1 | 0.221 | 0.075 | 2.934 | 0.003 | 0.221 | 0.315 |
| total2 | 0.220 | 0.076 | 2.897 | 0.004 | 0.220 | 0.312 |
| total3 | 0.225 | 0.077 | 2.899 | 0.004 | 0.225 | 0.319 |
| total4 | 0.218 | 0.076 | 2.868 | 0.004 | 0.218 | 0.310 |
| total5 | 0.229 | 0.078 | 2.933 | 0.003 | 0.229 | 0.326 |
| total6 | 0.073 | 0.049 | 1.509 | 0.131 | 0.073 | 0.127 |
| total7 | 0.070 | 0.049 | 1.449 | 0.147 | 0.070 | 0.122 |
| total8 | 0.071 | 0.049 | 1.449 | 0.147 | 0.071 | 0.123 |
| total9 | 0.068 | 0.049 | 1.383 | 0.167 | 0.068 | 0.117 |
| total10 | 0.076 | 0.050 | 1.523 | 0.128 | 0.076 | 0.132 |
| total11 | 0.007 | 0.066 | 0.107 | 0.915 | 0.007 | 0.007 |
| total12 | 0.000 | 0.064 | 0.004 | 0.997 | 0.000 | 0.000 |
| total13 | -0.002 | 0.064 | -0.033 | 0.974 | -0.002 | -0.002 |
| total14 | -0.001 | 0.066 | -0.014 | 0.989 | -0.001 | -0.001 |
| total15 | 0.005 | 0.063 | 0.083 | 0.934 | 0.005 | 0.005 |
|  |  |  |  |  |  |  |

### Second Timepoint

| **Suppl. file 1f. Complete outcome of structural equation with COVID-19 related life concerns from second survey timepoint.** | | | | | | |
| --- | --- | --- | --- | --- | --- | --- |
| **Estimator** | **ML** |  |  |  |  |  |
| Optimization method | NLMINB |  |  |  |  |  |
| Number of free parameters | 108 |  |  |  |  |  |
|  | Used | Total |  |  |  |  |
| Number of observations | 431 | 464 |  |  |  |  |
| **Model Test User Model:** |  |  |  |  |  |  |
| Test statistic | 440.014 |  |  |  |  |  |
| Degrees of freedom | 135 |  |  |  |  |  |
| P-value (Chi-square) | 0.000 |  |  |  |  |  |
| **Model Test Baseline Model:** |  |  |  |  |  |  |
| Test statistic | 2.430.458 |  |  |  |  |  |
| Degrees of freedom | 225 |  |  |  |  |  |
| P-value | 0.000 |  |  |  |  |  |
| **User Model versus Baseline Model:** |  |  |  |  |  |  |
| Comparative Fit Index (CFI) | 0.862 |  |  |  |  |  |
| Tucker-Lewis Index (TLI) | 0.770 |  |  |  |  |  |
| **Loglikelihood and Information Criteria:** |  |  |  |  |  |  |
| Loglikelihood user model (H0) | -8403.637 |  |  |  |  |  |
| Loglikelihood unrestricted model (H1) | -8183.630 |  |  |  |  |  |
| Akaike (AIC) | 17023.274 | |  |  |  |  |
| Bayesian (BIC) | 17462.414 | |  |  |  |  |
| Sample-size adjusted Bayesian (BIC) | 17119.684 | |  |  |  |  |
| **Root Mean Square Error of Approximation:** | |  |  |  |  |  |
| RMSEA | 0.072 |  |  |  |  |  |
| 90 Percent confidence interval - lower | 0.065 |  |  |  |  |  |
| 90 Percent confidence interval - upper | 0.080 |  |  |  |  |  |
| P-value RMSEA <= 0.05 | 0.000 |  |  |  |  |  |
| **Standardized Root Mean Square Residual:** | | |  |  |  |  |
| SRMR | 0.063 |  |  |  |  |  |
| **Parameter Estimates:** |  |  |  |  |  |  |
| Standard errors | Bootstrap |  |  |  |  |  |
| Number of requested bootstrap draws | 1000 |  |  |  |  |  |
| Number of successful bootstrap draws | 1000 |  |  |  |  |  |
| **Latent Variables:** |  |  |  |  |  |  |
|  | Estimate | Std.Err | z-value | P(>\|z\|) | Std.lv | Std.all |
| **Living Conditions =~** |  |  |  |  |  |  |
| Number_rooms | 1.000 |  |  |  | 1.988 | 0.905 |
| Number people per household | 0.412 | 0.035 | 11.667 | 0.000 | 0.819 | 0.647 |
| Living Area | 0.344 | 0.041 | 8.287 | 0.000 | 0.683 | 0.498 |
| Garden_yard | 0.134 | 0.017 | 8.018 | 0.000 | 0.267 | 0.569 |
| **Health before COVID19 =~** | |  |  |  |  |  |
| Regular treatment physical illness | 1.000 |  |  |  | 0.159 | 0.496 |
| Mental health status before COVID19 | -5.172 | 1.110 | -4.661 | 0.000 | -0.824 | -0.803 |
| Physical health status before COVID19 | -3.316 | 0.679 | -4.880 | 0.000 | -0.528 | -0.573 |
| **COVID19 related life concerns =~** | |  |  |  |  |  |
| Financial impact due to COVID19 | 0.602 | 0.065 | 9.220 | 0.000 | 0.602 | 0.628 |
| Concerns about life stability | 0.912 | 0.078 | 11.727 | 0.000 | 0.912 | 0.813 |
| Restrictions perceived as stressful | 0.453 | 0.065 | 6.973 | 0.000 | 0.453 | 0.437 |
|  |  |  |  |  |  |  |
| **Regressions:** |  |  |  |  |  |  |
|  | Estimate | Std.Err | z-value | P(>\|z\|) | Std.lv | Std.all |
| **depression ~** |  |  |  |  |  |  |
| Health before COVID19 | -1.724 | 0.358 | -4.810 | 0.000 | -0.275 | -0.412 |
| Country (residence) | -0.277 | 0.068 | -4.061 | 0.000 | -0.277 | -0.174 |
| Age | -0.006 | 0.002 | -3.793 | 0.000 | -0.006 | -0.144 |
| Gender | -0.009 | 0.061 | -0.146 | 0.884 | -0.009 | -0.006 |
| Highest education | 0.012 | 0.021 | 0.553 | 0.580 | 0.012 | 0.022 |
| Living Conditions | -0.003 | 0.015 | -0.216 | 0.829 | -0.007 | -0.010 |
| **anxiety ~** |  |  |  |  |  |  |
| Health before COVID19 | -1.390 | 0.254 | -5.476 | 0.000 | -0.221 | -0.406 |
| Country (residence) | -0.163 | 0.065 | -2.496 | 0.013 | -0.163 | -0.125 |
| Age | -0.008 | 0.001 | -5.806 | 0.000 | -0.008 | -0.220 |
| Gender | 0.019 | 0.048 | 0.384 | 0.701 | 0.019 | 0.015 |
| Highest education | -0.040 | 0.020 | -2.033 | 0.042 | -0.040 | -0.090 |
| Living Conditions | 0.012 | 0.014 | 0.829 | 0.407 | 0.023 | 0.043 |
| **SPQ_total ~** |  |  |  |  |  |  |
| Health before COVID19 | -2.861 | 0.534 | -5.353 | 0.000 | -0.456 | -0.466 |
| Country (residence) | -0.034 | 0.118 | -0.286 | 0.775 | -0.034 | -0.014 |
| Age | -0.006 | 0.002 | -2.606 | 0.009 | -0.006 | -0.100 |
| Gender | -0.213 | 0.093 | -2.284 | 0.022 | -0.213 | -0.096 |
| Highest education | -0.119 | 0.034 | -3.482 | 0.000 | -0.119 | -0.150 |
| Living Conditions | 0.020 | 0.026 | 0.788 | 0.430 | 0.040 | 0.041 |
| **Media ~** |  |  |  |  |  |  |
| Health before COVID19 | -0.272 | 0.161 | -1.695 | 0.090 | -0.043 | -0.105 |
| Country (residence) | -0.122 | 0.047 | -2.605 | 0.009 | -0.122 | -0.123 |
| Age | -0.004 | 0.001 | -2.911 | 0.004 | -0.004 | -0.154 |
| Gender | -0.039 | 0.043 | -0.912 | 0.362 | -0.039 | -0.042 |
| Highest education | -0.080 | 0.015 | -5.174 | 0.000 | -0.080 | -0.239 |
| Living Conditions | -0.007 | 0.011 | -0.645 | 0.519 | -0.014 | -0.035 |
| **Drugs ~** |  |  |  |  |  |  |
| Health before COVID19 | -0.256 | 0.152 | -1.679 | 0.093 | -0.041 | -0.119 |
| Country (residence) | -0.103 | 0.047 | -2.172 | 0.030 | -0.103 | -0.126 |
| Age | -0.000 | 0.001 | -0.366 | 0.714 | -0.000 | -0.016 |
| Gender | 0.029 | 0.035 | 0.850 | 0.395 | 0.029 | 0.038 |
| Highest education | -0.032 | 0.015 | -2.045 | 0.041 | -0.032 | -0.114 |
| Living Conditions | -0.021 | 0.008 | -2.465 | 0.014 | -0.041 | -0.120 |
| **Alcohol ~** |  |  |  |  |  |  |
| Health before COVID19 | 1.471 | 0.915 | 1.608 | 0.108 | 0.234 | 0.109 |
| Country (residence) | -0.506 | 0.247 | -2.046 | 0.041 | -0.506 | -0.099 |
| Age | 0.018 | 0.007 | 2.665 | 0.008 | 0.018 | 0.134 |
| Gender | -0.462 | 0.236 | -1.955 | 0.051 | -0.462 | -0.095 |
| Highest education | 0.166 | 0.093 | 1.772 | 0.076 | 0.166 | 0.095 |
| Living Conditions | 0.042 | 0.058 | 0.720 | 0.471 | 0.083 | 0.039 |
| **Exercise ~** | |  |  |  |  |  |
| Health before COVID19 | 1.044 | 0.443 | 2.355 | 0.019 | 0.166 | 0.164 |
| Country (residence) | -0.186 | 0.126 | -1.472 | 0.141 | -0.186 | -0.077 |
| Age | 0.000 | 0.003 | 0.042 | 0.967 | 0.000 | 0.002 |
| Gender | -0.165 | 0.115 | -1.438 | 0.150 | -0.165 | -0.072 |
| Highest education | 0.055 | 0.042 | 1.308 | 0.191 | 0.055 | 0.067 |
| Living Conditions | -0.010 | 0.027 | -0.369 | 0.712 | -0.020 | -0.019 |
| **Sleep during week ~** | |  |  |  |  |  |
| Health before COVID19 | 0.397 | 0.153 | 2.588 | 0.010 | 0.063 | 0.183 |
| Country (residence) | 0.070 | 0.042 | 1.664 | 0.096 | 0.070 | 0.085 |
| Age | -0.001 | 0.001 | -1.356 | 0.175 | -0.001 | -0.061 |
| Gender | 0.061 | 0.041 | 1.472 | 0.141 | 0.061 | 0.078 |
| Highest education | 0.020 | 0.016 | 1.289 | 0.197 | 0.020 | 0.072 |
| Living Conditions | -0.003 | 0.010 | -0.360 | 0.719 | -0.007 | -0.020 |
| **healthBeforeCo19 ~** | |  |  |  |  |  |
| Country (residence) | 0.085 | 0.028 | 3.052 | 0.002 | 0.534 | 0.224 |
| Age | 0.001 | 0.001 | 1.598 | 0.110 | 0.006 | 0.089 |
| Gender | 0.013 | 0.019 | 0.654 | 0.513 | 0.079 | 0.035 |
| Highest education | 0.015 | 0.010 | 1.572 | 0.116 | 0.095 | 0.117 |
| Living Conditions | 0.017 | 0.007 | 2.573 | 0.010 | 0.217 | 0.217 |
| **depression ~** |  |  |  |  |  |  |
| COVID19 related life concerns (c1) | 0.278 | 0.044 | 6.352 | 0.000 | 0.278 | 0.417 |
| **anxiety ~** |  |  |  |  |  |  |
| COVID19 related life concerns (c2) | 0.183 | 0.047 | 3.899 | 0.000 | 0.183 | 0.336 |
| **SPQ_total ~** |  |  |  |  |  |  |
| COVID19 related life concerns (c3) | 0.225 | 0.066 | 3.400 | 0.001 | 0.225 | 0.230 |
| **Media ~** |  |  |  |  |  |  |
| COVID19 related life concerns (a1) | 0.005 | 0.024 | 0.217 | 0.828 | 0.005 | 0.012 |
| **Drugs ~** |  |  |  |  |  |  |
| COVID19 related life concerns (a2) | 0.038 | 0.023 | 1.650 | 0.099 | 0.038 | 0.110 |
| **Alcohol ~** |  |  |  |  |  |  |
| COVID19 related life concerns (a3) | -0.041 | 0.137 | -0.300 | 0.764 | -0.041 | -0.019 |
| **Exercise ~** | |  |  |  |  |  |
| COVID19 related life concerns (a4) | -0.099 | 0.066 | -1.503 | 0.133 | -0.099 | -0.097 |
| **Sleep during week ~** | |  |  |  |  |  |
| COVID19 related life concerns (a5) | -0.022 | 0.023 | -0.969 | 0.332 | -0.022 | -0.063 |
| **depression ~** |  |  |  |  |  |  |
| Media (b1) | 0.099 | 0.065 | 1.523 | 0.128 | 0.099 | 0.061 |
| Drugs (b2) | 0.140 | 0.115 | 1.219 | 0.223 | 0.140 | 0.072 |
| Alcohol (b3) | 0.020 | 0.013 | 1.489 | 0.137 | 0.020 | 0.063 |
| Exercise (b4) | -0.015 | 0.025 | -0.606 | 0.544 | -0.015 | -0.023 |
| Sleep during week(b5) | -0.189 | 0.083 | -2.272 | 0.023 | -0.189 | -0.098 |
| **anxiety ~** |  |  |  |  |  |  |
| Media (b6) | 0.105 | 0.054 | 1.942 | 0.052 | 0.105 | 0.080 |
| Drug (b7) | 0.110 | 0.089 | 1.233 | 0.218 | 0.110 | 0.069 |
| Alcohol (b8) | 0.002 | 0.011 | 0.181 | 0.857 | 0.002 | 0.008 |
| Exercise (b9) | 0.018 | 0.023 | 0.792 | 0.428 | 0.018 | 0.034 |
| Sleep during week (b10) | -0.130 | 0.072 | -1.792 | 0.073 | -0.130 | -0.082 |
| **SPQ_total ~** |  |  |  |  |  |  |
| Media (b11) | 0.255 | 0.101 | 2.532 | 0.011 | 0.255 | 0.108 |
| Drug (b12) | 0.138 | 0.132 | 1.048 | 0.295 | 0.138 | 0.048 |
| Alcohol (b13) | 0.013 | 0.019 | 0.706 | 0.480 | 0.013 | 0.030 |
| Exercise (b14) | 0.031 | 0.044 | 0.708 | 0.479 | 0.031 | 0.032 |
| Sleep during week (b15) | -0.300 | 0.124 | -2.428 | 0.015 | -0.300 | -0.106 |
|  |  |  |  |  |  |  |
| **Covariances:** |  |  |  |  |  |  |
|  | Estimate | Std.Err | z-value | P(>\|z\|) | Std.lv | Std.all |
| .anxiety ~~ |  |  |  |  |  |  |
| .SPQ_total | 0.121 | 0.024 | 5.041 | 0.000 | 0.121 | 0.416 |
| .depression ~~ |  |  |  |  |  |  |
| .SPQ_total | 0.076 | 0.027 | 2.791 | 0.005 | 0.076 | 0.225 |
| .anxiety | 0.088 | 0.017 | 5.181 | 0.000 | 0.088 | 0.495 |
| Living Conditions ~~ |  |  |  |  |  |  |
| COVID19 related life concerns | -0.176 | 0.123 | -1.422 | 0.155 | -0.088 | -0.088 |
|  |  |  |  |  |  |  |
| **Variances:** |  |  |  |  |  |  |
|  | Estimate | Std.Err | z-value | P(>\|z\|) | Std.lv | Std.all |
| COVID19 related life concerns | 1.000 |  |  |  | 1.000 | 1.000 |
| .Number rooms | 0.870 | 0.309 | 2.819 | 0.005 | 0.870 | 0.180 |
| .Number people per household | 0.928 | 0.076 | 12.292 | 0.000 | 0.928 | 0.581 |
| .Living Area | 1.412 | 0.092 | 15.393 | 0.000 | 1.412 | 0.752 |
| .Garden_yard | 0.149 | 0.010 | 14.352 | 0.000 | 0.149 | 0.677 |
| .Regular treatment physical illness | 0.078 | 0.008 | 9.167 | 0.000 | 0.078 | 0.754 |
| .Mental health status before COVID19 | 0.374 | 0.092 | 4.068 | 0.000 | 0.374 | 0.356 |
| .Physical health status before COVID19 | 0.571 | 0.057 | 10.019 | 0.000 | 0.571 | 0.672 |
| .Financial Impact due to COVID19 | 0.557 | 0.063 | 8.874 | 0.000 | 0.557 | 0.606 |
| .Concerns about life stability | 0.426 | 0.109 | 3.915 | 0.000 | 0.426 | 0.339 |
| .Restrictions perceived as stressful | 0.868 | 0.067 | 12.934 | 0.000 | 0.868 | 0.809 |
| .depression | 0.206 | 0.028 | 7.301 | 0.000 | 0.206 | 0.464 |
| .anxiety | 0.155 | 0.020 | 7.892 | 0.000 | 0.155 | 0.521 |
| .SPQ_total | 0.550 | 0.047 | 11.603 | 0.000 | 0.550 | 0.576 |
| .Media | 0.149 | 0.010 | 182 | 0.000 | 0.149 | 0.873 |
| .Drug | 0.106 | 0.011 | 9.588 | 0.000 | 0.106 | 0.910 |
| .Alcohol | 4.300 | 0.169 | 25.434 | 0.000 | 4.300 | 0.936 |
| .Exercise | 0.981 | 0.072 | 13.698 | 0.000 | 0.981 | 0.951 |
| .Sleep during week | 0.112 | 0.011 | 10.198 | 0.000 | 0.112 | 0.935 |
| Living Conditions | 3.953 | 0.475 | 8.319 | 0.000 | 1.000 | 1.000 |
| .health before COVID19 | 0.022 | 0.006 | 3.490 | 0.000 | 0.879 | 0.879 |
|  |  |  |  |  |  |  |
| **Defined Parameters:** |  |  |  |  |  |  |
|  | Estimate | Std.Err | z-value | P(>\|z\|) | Std.lv | Std.all |
| indirect1 | 0.001 | 0.003 | 0.188 | 0.851 | 0.001 | 0.001 |
| indirect2 | 0.005 | 0.006 | 0.877 | 0.381 | 0.005 | 0.008 |
| indirect3 | -0.001 | 0.003 | -0.243 | 0.808 | -0.001 | -0.001 |
| indirect4 | 0.001 | 0.003 | 0.506 | 0.613 | 0.001 | 0.002 |
| indirect5 | 0.004 | 0.005 | 0.855 | 0.392 | 0.004 | 0.006 |
| indirect6 | 0.001 | 0.003 | 0.197 | 0.844 | 0.001 | 0.001 |
| indirect7 | 0.004 | 0.005 | 0.870 | 0.384 | 0.004 | 0.008 |
| indirect8 | -0.000 | 0.002 | -0.049 | 0.961 | -0.000 | -0.000 |
| indirect9 | -0.002 | 0.003 | -0.613 | 0.540 | -0.002 | -0.003 |
| indirect10 | 0.003 | 0.004 | 0.798 | 0.425 | 0.003 | 0.005 |
| indirect11 | 0.001 | 0.007 | 0.201 | 0.840 | 0.001 | 0.001 |
| indirect12 | 0.005 | 0.007 | 0.776 | 0.437 | 0.005 | 0.005 |
| indirect13 | -0.001 | 0.003 | -0.161 | 0.872 | -0.001 | -0.001 |
| indirect14 | -0.003 | 0.006 | -0.540 | 0.589 | -0.003 | -0.003 |
| indirect15 | 0.007 | 0.007 | 0.898 | 0.369 | 0.007 | 0.007 |
| total1 | 0.278 | 0.044 | 6.350 | 0.000 | 0.278 | 0.418 |
| total2 | 0.283 | 0.045 | 6.347 | 0.000 | 0.283 | 0.425 |
| total3 | 0.277 | 0.044 | 6.227 | 0.000 | 0.277 | 0.416 |
| total4 | 0.279 | 0.043 | 6.432 | 0.000 | 0.279 | 0.419 |
| total5 | 0.282 | 0.044 | 6.388 | 0.000 | 0.282 | 0.423 |
| total6 | 0.183 | 0.047 | 3.924 | 0.000 | 0.183 | 0.337 |
| total7 | 0.187 | 0.047 | 3.957 | 0.000 | 0.187 | 0.343 |
| total8 | 0.183 | 0.047 | 3.906 | 0.000 | 0.183 | 0.335 |
| total9 | 0.181 | 0.046 | 3.899 | 0.000 | 0.181 | 0.332 |
| total10 | 0.186 | 0.047 | 3.914 | 0.000 | 0.186 | 0.341 |
| total11 | 0.226 | 0.066 | 3.408 | 0.001 | 0.226 | 0.231 |
| total12 | 0.230 | 0.066 | 3.480 | 0.001 | 0.230 | 0.235 |
| total13 | 0.224 | 0.066 | 3.397 | 0.001 | 0.224 | 0.229 |
| total14 | 0.222 | 0.066 | 3.384 | 0.001 | 0.222 | 0.227 |
| total15 | 0.231 | 0.066 | 3.492 | 0.000 | 0.231 | 0.237 |
|  |  |  |  |  |  |  |

### Third timepoint

| **Suppl. file 1g. Complete outcome of structural equation with COVID-19 related life concerns from third survey timepoint.** | | | | | | |
| --- | --- | --- | --- | --- | --- | --- |
| **Estimator** | **ML** |  |  |  |  |  |
| Optimization method | NLMINB |  |  |  |  |  |
| Number of free parameters | 108 |  |  |  |  |  |
|  | Used | Total |  |  |  |  |
| Number of observations | 496 | 532 |  |  |  |  |
|  |  |  |  |  |  |  |
| **Model Test User Model:** |  |  |  |  |  |  |
| Test statistic | 426.425 |  |  |  |  |  |
| Degrees of freedom | 135 |  |  |  |  |  |
| P-value (Chi-square) | 0.000 |  |  |  |  |  |
| **Model Test Baseline Model:** |  |  |  |  |  |  |
| Test statistic | 2.725.462 |  |  |  |  |  |
| Degrees of freedom | 225 |  |  |  |  |  |
| P-value | 0.000 |  |  |  |  |  |
| **User Model versus Baseline Model:** |  |  |  |  |  |  |
| Comparative Fit Index (CFI) | 0.883 |  |  |  |  |  |
| Tucker-Lewis Index (TLI) | 0.806 |  |  |  |  |  |
| **Loglikelihood and Information Criteria:** |  |  |  |  |  |  |
| Loglikelihood user model (H0) | -10554.934 | |  |  |  |  |
| Loglikelihood unrestricted model (H1) | -10341.722 | |  |  |  |  |
| Akaike (AIC) | 21325.868 | |  |  |  |  |
| Bayesian (BIC) | 21780.178 | |  |  |  |  |
| Sample-size adjusted Bayesian (BIC) | 21437.383 | |  |  |  |  |
| **Root Mean Square Error of Approximation:** | | |  |  |  |  |
| RMSEA | 0.066 |  |  |  |  |  |
| 90 Percent confidence interval - lower | 0.059 |  |  |  |  |  |
| 90 Percent confidence interval - upper | 0.073 |  |  |  |  |  |
| P-value RMSEA <= 0.05 | 0.000 |  |  |  |  |  |
| **Standardized Root Mean Square Residual:** |  |  |  |  |  |  |
| SRMR | 0.056 |  |  |  |  |  |
| **Parameter Estimates:** |  |  |  |  |  |  |
| Standard errors | Bootstrap |  |  |  |  |  |
| Number of requested bootstrap draws | 1000 |  |  |  |  |  |
| Number of successful bootstrap draws | 1000 |  |  |  |  |  |
| **Latent Variables:** |  |  |  |  |  |  |
|  | Estimate | Std.Err | z-value | P(>\|z\|) | Std.lv | Std.all |
| **Living Conditions =~** |  |  |  |  |  |  |
| Number_rooms | 1.000 |  |  |  | 1.744 | 0.804 |
| Number people per household | 0.402 | 0.041 | 9.927 | 0.000 | 0.702 | 0.531 |
| Living Area | 0.424 | 0.062 | 6.815 | 0.000 | 0.739 | 0.535 |
| Garden_yard | 0.159 | 0.023 | 6.783 | 0.000 | 0.278 | 0.605 |
| **Health before COVID19 =~** | |  |  |  |  |  |
| Regular treatment physical illness | 1.000 |  |  |  | 0.205 | 0.566 |
| Mental health status before COVID19 | -4.338 | 0.614 | -7.069 | 0.000 | -0.889 | -0.867 |
| Physical health status before COVID19 | -2.995 | 0.416 | -7.207 | 0.000 | -0.614 | -0.619 |
| **COVID19 related life concerns =~** | |  |  |  |  |  |
| Financial impact due to COVID19 | 0.672 | 0.066 | 10.154 | 0.000 | 0.672 | 0.627 |
| Concerns about life stability | 0.997 | 0.062 | 16.127 | 0.000 | 0.997 | 0.772 |
| Restrictions perceived as stressful | 0.780 | 0.064 | 12.109 | 0.000 | 0.780 | 0.660 |
|  |  |  |  |  |  |  |
| **Regressions:** |  |  |  |  |  |  |
|  | Estimate | Std.Err | z-value | P(>\|z\|) | Std.lv | Std.all |
| **depression ~** |  |  |  |  |  |  |
| Health before COVID19 | -1.575 | 0.228 | -6.893 | 0.000 | -0.323 | -0.434 |
| Country (residence) | -0.189 | 0.069 | -2.755 | 0.006 | -0.189 | -0.117 |
| Age | -0.005 | 0.002 | -2.952 | 0.003 | -0.005 | -0.107 |
| Gender | 0.027 | 0.062 | 0.434 | 0.664 | 0.027 | 0.016 |
| Highest education | 0.020 | 0.019 | 1.038 | 0.299 | 0.020 | 0.039 |
| Living Conditions | 0.019 | 0.020 | 0.973 | 0.331 | 0.034 | 0.045 |
| **anxiety ~** |  |  |  |  |  |  |
| Health before COVID19 | -1.120 | 0.206 | -5.447 | 0.000 | -0.229 | -0.400 |
| Country (residence) | -0.140 | 0.063 | -2.226 | 0.026 | -0.140 | -0.112 |
| Age | -0.007 | 0.001 | -4.627 | 0.000 | -0.007 | -0.172 |
| Gender | 0.125 | 0.043 | 2.911 | 0.004 | 0.125 | 0.098 |
| Highest education | 0.011 | 0.017 | 0.678 | 0.498 | 0.011 | 0.030 |
| Living Conditions | 0.021 | 0.016 | 1.317 | 0.188 | 0.037 | 0.065 |
| **SPQ_total ~** |  |  |  |  |  |  |
| Health before COVID19 | -1.836 | 0.300 | -6.112 | 0.000 | -0.376 | -0.373 |
| Country (residence) | -0.083 | 0.109 | -0.761 | 0.446 | -0.083 | -0.038 |
| Age | -0.011 | 0.003 | -4.336 | 0.000 | -0.011 | -0.167 |
| Gender | 0.039 | 0.095 | 0.411 | 0.681 | 0.039 | 0.017 |
| Highest education | -0.065 | 0.028 | -2.286 | 0.022 | -0.065 | -0.096 |
| Living Conditions | -0.023 | 0.029 | -0.787 | 0.431 | -0.039 | -0.039 |
| **Media ~** |  |  |  |  |  |  |
| Health before COVID19 | -0.130 | 0.123 | -1.055 | 0.292 | -0.027 | -0.055 |
| Country (residence) | -0.059 | 0.044 | -1.331 | 0.183 | -0.059 | -0.056 |
| Age | -0.001 | 0.001 | -0.836 | 0.403 | -0.001 | -0.037 |
| Gender | -0.140 | 0.048 | -2.907 | 0.004 | -0.140 | -0.130 |
| Highest education | -0.071 | 0.014 | -5.258 | 0.000 | -0.071 | -0.218 |
| Living Conditions | -0.031 | 0.015 | -2.033 | 0.042 | -0.054 | -0.110 |
| **Drugs ~** |  |  |  |  |  |  |
| Health before COVID19 | -0.387 | 0.101 | -3.818 | 0.000 | -0.079 | -0.214 |
| Country (residence) | 0.054 | 0.038 | 1.393 | 0.164 | 0.054 | 0.067 |
| Age | -0.001 | 0.001 | -1.364 | 0.173 | -0.001 | -0.058 |
| Gender | 0.008 | 0.034 | 0.247 | 0.805 | 0.008 | 0.010 |
| Highest education | -0.004 | 0.011 | -0.346 | 0.729 | -0.004 | -0.016 |
| Living Conditions | -0.014 | 0.011 | -1.258 | 0.208 | -0.024 | -0.065 |
| **Alcohol ~** |  |  |  |  |  |  |
| Health before COVID19 | 1.292 | 0.567 | 2.279 | 0.023 | 0.265 | 0.119 |
| Country (residence) | -0.233 | 0.209 | -1.114 | 0.265 | -0.233 | -0.048 |
| Age | 0.022 | 0.007 | 3.212 | 0.001 | 0.022 | 0.147 |
| Gender | -0.237 | 0.232 | -1.022 | 0.307 | -0.237 | -0.048 |
| Highest education | 0.233 | 0.070 | 3.342 | 0.001 | 0.233 | 0.156 |
| Living Conditions | 0.012 | 0.063 | 0.183 | 0.855 | 0.020 | 0.009 |
| **Exercise ~** | |  |  |  |  |  |
| Health before COVID19 | -0.130 | 0.291 | -0.448 | 0.654 | -0.027 | -0.023 |
| Country (residence) | -0.201 | 0.116 | -1.737 | 0.082 | -0.201 | -0.080 |
| Age | 0.004 | 0.004 | 1.056 | 0.291 | 0.004 | 0.050 |
| Gender | 0.110 | 0.113 | 0.971 | 0.331 | 0.110 | 0.043 |
| Highest education | 0.142 | 0.035 | 4.023 | 0.000 | 0.142 | 0.183 |
| Living Conditions | 0.070 | 0.036 | 1.914 | 0.056 | 0.121 | 0.105 |
| **Sleep during week ~** | |  |  |  |  |  |
| Health before COVID19 | -0.065 | 0.189 | -0.341 | 0.733 | -0.013 | -0.021 |
| Country (residence) | 0.143 | 0.071 | 2.026 | 0.043 | 0.143 | 0.104 |
| Age | -0.004 | 0.002 | -2.324 | 0.020 | -0.004 | -0.105 |
| Gender | 0.264 | 0.058 | 4.532 | 0.000 | 0.264 | 0.188 |
| Highest education | 0.008 | 0.018 | 0.468 | 0.640 | 0.008 | 0.019 |
| Living Conditions | -0.016 | 0.020 | -0.832 | 0.405 | -0.028 | -0.045 |
| **healthBeforeCo19 ~** | |  |  |  |  |  |
| Country (residence) | 0.050 | 0.026 | 1.929 | 0.054 | 0.245 | 0.113 |
| Age | 0.001 | 0.001 | 2.203 | 0.028 | 0.007 | 0.107 |
| Gender | -0.022 | 0.022 | -1.016 | 0.310 | -0.109 | -0.049 |
| Highest education | 0.002 | 0.007 | 0.235 | 0.814 | 0.008 | 0.012 |
| Living Conditions | 0.011 | 0.009 | 1.260 | 0.208 | 0.092 | 0.092 |
| **depression ~** |  |  |  |  |  |  |
| COVID19 related life concerns (c1) | 0.305 | 0.048 | 6.387 | 0.000 | 0.305 | 0.409 |
| **anxiety ~** |  |  |  |  |  |  |
| COVID19 related life concerns (c2) | 0.144 | 0.036 | 3.987 | 0.000 | 0.144 | 0.252 |
| **SPQ_total ~** |  |  |  |  |  |  |
| COVID19 related life concerns (c3) | 0.090 | 0.060 | 1.510 | 0.131 | 0.090 | 0.089 |
| **Media ~** |  |  |  |  |  |  |
| COVID19 related life concerns (a1) | 0.099 | 0.026 | 3.850 | 0.000 | 0.099 | 0.203 |
| **Drugs ~** |  |  |  |  |  |  |
| COVID19 related life concerns (a2) | 0.091 | 0.025 | 3.693 | 0.000 | 0.091 | 0.246 |
| **Alcohol ~** |  |  |  |  |  |  |
| COVID19 related life concerns (a3) | 0.075 | 0.120 | 0.621 | 0.535 | 0.075 | 0.034 |
| **Exercise ~** | |  |  |  |  |  |
| COVID19 related life concerns (a4) | -0.107 | 0.057 | -1.887 | 0.059 | -0.107 | -0.093 |
| **Sleep during week ~** | |  |  |  |  |  |
| COVID19 related life concerns (a5) | -0.168 | 0.033 | -5.099 | 0.000 | -0.168 | -0.264 |
| **depression ~** |  |  |  |  |  |  |
| Media (b1) | 0.189 | 0.062 | 3.054 | 0.002 | 0.189 | 0.124 |
| Drugs (b2) | 0.140 | 0.101 | 1.386 | 0.166 | 0.140 | 0.070 |
| Alcohol (b3) | 0.020 | 0.012 | 1.653 | 0.098 | 0.020 | 0.061 |
| Exercise (b4) | -0.025 | 0.024 | -1.049 | 0.294 | -0.025 | -0.039 |
| Sleep during week(b5) | -0.046 | 0.050 | -0.934 | 0.350 | -0.046 | -0.040 |
| **anxiety ~** |  |  |  |  |  |  |
| Media (b6) | 0.174 | 0.051 | 3.406 | 0.001 | 0.174 | 0.148 |
| Drug (b7) | 0.143 | 0.082 | 1.731 | 0.083 | 0.143 | 0.092 |
| Alcohol (b8) | -0.009 | 0.011 | -0.767 | 0.443 | -0.009 | -0.034 |
| Exercise (b9) | 0.009 | 0.019 | 0.503 | 0.615 | 0.009 | 0.019 |
| Sleep during week (b10) | -0.005 | 0.049 | -0.107 | 0.915 | -0.005 | -0.006 |
| **SPQ_total ~** |  |  |  |  |  |  |
| Media (b11) | 0.306 | 0.089 | 3.444 | 0.001 | 0.306 | 0.148 |
| Drug (b12) | 0.114 | 0.136 | 0.842 | 0.400 | 0.114 | 0.042 |
| Alcohol (b13) | -0.020 | 0.020 | -0.995 | 0.320 | -0.020 | -0.043 |
| Exercise (b14) | 0.007 | 0.035 | 0.187 | 0.852 | 0.007 | 0.007 |
| Sleep during week (b15) | -0.145 | 0.076 | -1.906 | 0.057 | -0.145 | -0.091 |
|  |  |  |  |  |  |  |
| **Covariances:** |  |  |  |  |  |  |
|  | Estimate | Std.Err | z-value | P(>\|z\|) | Std.lv | Std.all |
| .anxiety ~~ |  |  |  |  |  |  |
| .SPQ_total | 0.209 | 0.028 | 7.382 | 0.000 | 0.209 | 0.550 |
| .depression ~~ |  |  |  |  |  |  |
| .SPQ_total | 0.178 | 0.029 | 6.099 | 0.000 | 0.178 | 0.394 |
| .anxiety | 0.131 | 0.020 | 6.710 | 0.000 | 0.131 | 0.553 |
| Living Conditions ~~ |  |  |  |  |  |  |
| COVID19 related life concerns | -0.094 | 0.116 | -0.811 | 0.418 | -0.054 | -0.054 |
|  |  |  |  |  |  |  |
| **Variances:** |  |  |  |  |  |  |
|  | Estimate | Std.Err | z-value | P(>\|z\|) | Std.lv | Std.all |
| COVID19 related life concerns | 1.000 |  |  |  | 1.000 | 1.000 |
| .Number rooms | 1.667 | 0.364 | 4.574 | 0.000 | 1.667 | 0.354 |
| .Number people per household | 1.253 | 0.150 | 8.375 | 0.000 | 1.253 | 0.718 |
| .Living Area | 1.362 | 0.112 | 12.187 | 0.000 | 1.362 | 0.714 |
| .Garden_yard | 0.134 | 0.012 | 11.147 | 0.000 | 0.134 | 0.634 |
| .Regular treatment physical illness | 0.089 | 0.009 | 9.835 | 0.000 | 0.089 | 0.680 |
| .Mental health status before COVID19 | 0.260 | 0.072 | 3.615 | 0.000 | 0.260 | 0.248 |
| .Physical health status before COVID19 | 0.607 | 0.051 | 12.012 | 0.000 | 0.607 | 0.617 |
| .Financial Impact due to COVID19 | 0.698 | 0.068 | 10.314 | 0.000 | 0.698 | 0.607 |
| .Concerns about life stability | 0.673 | 0.103 | 6.518 | 0.000 | 0.673 | 0.404 |
| .Restrictions perceived as stressful | 0.787 | 0.091 | 8.609 | 0.000 | 0.787 | 0.564 |
| .depression | 0.282 | 0.028 | 10.157 | 0.000 | 0.282 | 0.510 |
| .anxiety | 0.200 | 0.023 | 8.669 | 0.000 | 0.200 | 0.605 |
| .SPQ_total | 0.724 | 0.059 | 12.251 | 0.000 | 0.724 | 0.711 |
| .Media | 0.208 | 0.008 | 26.634 | 0.000 | 0.208 | 0.878 |
| .Drug | 0.121 | 0.009 | 12.953 | 0.000 | 0.121 | 0.877 |
| .Alcohol | 4.586 | 0.161 | 28.528 | 0.000 | 4.586 | 0.926 |
| .Exercise | 1.238 | 0.077 | 16.139 | 0.000 | 1.238 | 0.933 |
| .Sleep during week | 0.352 | 0.026 | 13.710 | 0.000 | 0.352 | 0.876 |
| Living Conditions | 3.042 | 0.457 | 6.651 | 0.000 | 1.000 | 1.000 |
| .health before COVID19 | 0.040 | 0.009 | 4.546 | 0.000 | 0.963 | 0.963 |
|  |  |  |  |  |  |  |
| **Defined Parameters:** |  |  |  |  |  |  |
|  | Estimate | Std.Err | z-value | P(>\|z\|) | Std.lv | Std.all |
| indirect1 | 0.019 | 0.007 | 2.561 | 0.010 | 0.019 | 0.025 |
| indirect2 | 0.013 | 0.009 | 1.353 | 0.176 | 0.013 | 0.017 |
| indirect3 | 0.002 | 0.003 | 0.531 | 0.596 | 0.002 | 0.002 |
| indirect4 | 0.003 | 0.003 | 0.881 | 0.378 | 0.003 | 0.004 |
| indirect5 | 0.008 | 0.009 | 0.894 | 0.371 | 0.008 | 0.010 |
| indirect6 | 0.017 | 0.007 | 2.643 | 0.008 | 0.017 | 0.030 |
| indirect7 | 0.013 | 0.008 | 1.550 | 0.121 | 0.013 | 0.023 |
| indirect8 | -0.001 | 0.002 | -0.337 | 0.736 | -0.001 | -0.001 |
| indirect9 | -0.001 | 0.002 | -0.419 | 0.675 | -0.001 | -0.002 |
| indirect10 | 0.001 | 0.008 | 0.106 | 0.916 | 0.001 | 0.002 |
| indirect11 | 0.030 | 0.012 | 2.433 | 0.015 | 0.030 | 0.030 |
| indirect12 | 0.010 | 0.013 | 0.789 | 0.430 | 0.010 | 0.010 |
| indirect13 | -0.001 | 0.004 | -0.384 | 0.701 | -0.001 | -0.001 |
| indirect14 | -0.001 | 0.004 | -0.162 | 0.871 | -0.001 | -0.001 |
| indirect15 | 0.024 | 0.014 | 1.746 | 0.081 | 0.024 | 0.024 |
| total1 | 0.323 | 0.047 | 6.852 | 0.000 | 0.323 | 0.434 |
| total2 | 0.317 | 0.045 | 7.065 | 0.000 | 0.317 | 0.426 |
| total3 | 0.306 | 0.048 | 6.362 | 0.000 | 0.306 | 0.411 |
| total4 | 0.307 | 0.047 | 6.490 | 0.000 | 0.307 | 0.413 |
| total5 | 0.312 | 0.045 | 6.993 | 0.000 | 0.312 | 0.420 |
| total6 | 0.162 | 0.036 | 4.459 | 0.000 | 0.162 | 0.282 |
| total7 | 0.157 | 0.036 | 4.386 | 0.000 | 0.157 | 0.274 |
| total8 | 0.144 | 0.036 | 3.975 | 0.000 | 0.144 | 0.251 |
| total9 | 0.143 | 0.036 | 3.975 | 0.000 | 0.143 | 0.250 |
| total10 | 0.145 | 0.033 | 4.351 | 0.000 | 0.145 | 0.253 |
| total11 | 0.120 | 0.059 | 2.050 | 0.040 | 0.120 | 0.119 |
| total12 | 0.101 | 0.057 | 1.767 | 0.077 | 0.101 | 0.100 |
| total13 | 0.089 | 0.060 | 1.488 | 0.137 | 0.089 | 0.088 |
| total14 | 0.089 | 0.059 | 1.503 | 0.133 | 0.089 | 0.089 |
| total15 | 0.114 | 0.056 | 2.036 | 0.042 | 0.114 | 0.113 |
|  |  |  |  |  |  |  |

### Fourth timepoint

| **Suppl. file 1h. Complete outcome of structural equation with COVID-19 related life concerns from fourth survey timepoint.** | | | | | | |
| --- | --- | --- | --- | --- | --- | --- |
| **Estimator** | **ML** |  |  |  |  |  |
| Optimization method | NLMINB |  |  |  |  |  |
| Number of free parameters | 108 |  |  |  |  |  |
|  | Used | Total |  |  |  |  |
| Number of observations | 452 | 478 |  |  |  |  |
| **Model Test User Model:** |  |  |  |  |  |  |
| Test statistic | 413528 |  |  |  |  |  |
| Degrees of freedom | 135 |  |  |  |  |  |
| P-value (Chi-square) | 0.000 |  |  |  |  |  |
| **Model Test Baseline Model:** |  |  |  |  |  |  |
| Test statistic | 2315.281 |  |  |  |  |  |
| Degrees of freedom | 225 |  |  |  |  |  |
| P-value | 0.000 |  |  |  |  |  |
| **User Model versus Baseline Model:** |  |  |  |  |  |  |
| Comparative Fit Index (CFI) | 0.867 |  |  |  |  |  |
| Tucker-Lewis Index (TLI) | 0.778 |  |  |  |  |  |
| **Loglikelihood and Information Criteria:** |  |  |  |  |  |  |
| Loglikelihood user model (H0) | -9247.599 |  |  |  |  |  |
| Loglikelihood unrestricted model (H1) | -9040.835 |  |  |  |  |  |
| Akaike (AIC) | 18711.199 |  |  |  |  |  |
| Bayesian (BIC) | 19155.476 |  |  |  |  |  |
| Sample-size adjusted Bayesian (BIC) | 18812.723 |  |  |  |  |  |
| **Root Mean Square Error of Approximation:** |  |  |  |  |  |  |
| RMSEA | 0.068 |  |  |  |  |  |
| 90 Percent confidence interval - lower | 0.060 |  |  |  |  |  |
| 90 Percent confidence interval - upper | 0.075 |  |  |  |  |  |
| P-value RMSEA <= 0.05 | 0.000 |  |  |  |  |  |
| **Standardized Root Mean Square Residual:** |  |  |  |  |  |  |
| SRMR | 0.058 |  |  |  |  |  |
| **Parameter Estimates:** |  |  |  |  |  |  |
| Standard errors | Bootstrap |  |  |  |  |  |
| Number of requested bootstrap draws | 1000 |  |  |  |  |  |
| Number of successful bootstrap draws | 1000 |  |  |  |  |  |
| **Latent Variables:** |  |  |  |  |  |  |
|  | Estimate | Std.Err | z-value | P(>\|z\|) | Std.lv | Std.all |
| **Living Conditions** =~ |  |  |  |  |  |  |
| Number_rooms | 1.000 |  |  |  | 1.773 | 0.846 |
| Number people per household | 0.399 | 0.034 | 11.721 | 0.000 | 0.707 | 0.605 |
| Living Area | 0.346 | 0.051 | 6.763 | 0.000 | 0.613 | 0.456 |
| Garden_yard | 0.164 | 0.019 | 8.692 | 0.000 | 0.291 | 0.616 |
| **Health before COVID19** =~ | |  |  |  |  |  |
| Regular treatment physical illness | 1.000 |  |  |  | 0.145 | 0.434 |
| Mental health status before COVID19 | -5.618 | 1.279 | -4.392 | 0.000 | -0.816 | -0.813 |
| Physical health status before COVID19 | -3.995 | 0.877 | -4.553 | 0.000 | -0.580 | -0.633 |
| **COVID19 related life concerns** =~ | |  |  |  |  |  |
| Financial impact due to COVID19 | 0.577 | 0.072 | 7.957 | 0.000 | 0.577 | 0.583 |
| Concerns about life stability | 0.893 | 0.062 | 14.522 | 0.000 | 0.893 | 0.759 |
| Restrictions perceived as stressful | 0.740 | 0.066 | 11.254 | 0.000 | 0.740 | 0.601 |
|  |  |  |  |  |  |  |
| **Regressions:** |  |  |  |  |  |  |
|  | Estimate | Std.Err | z-value | P(>\|z\|) | Std.lv | Std.all |
| **depression** ~ | |  |  |  |  |  |
| Health before COVID19 | -2.027 | 0.461 | -4.401 | 0.000 | -0.294 | -0.394 |
| Country (residence) | -0.052 | 0.082 | -0.637 | 0.524 | -0.052 | -0.029 |
| Age | -0.004 | 0.002 | -2.154 | 0.031 | -0.004 | -0.082 |
| Gender | 0.004 | 0.075 | 0.058 | 0.954 | 0.004 | 0.002 |
| Highest education | -0.052 | 0.024 | -2.186 | 0.029 | -0.052 | -0.095 |
| Living Conditions | -0.010 | 0.020 | -0.476 | 0.634 | -0.017 | -0.023 |
| **anxiety** ~ |  |  |  |  |  |  |
| Health before COVID19 | -1.550 | 0.328 | -4.720 | 0.000 | -0.225 | -0.416 |
| Country (residence) | -0.154 | 0.064 | -2.429 | 0.015 | -0.154 | -0.117 |
| Age | -0.006 | 0.001 | -4.535 | 0.000 | -0.006 | -0.175 |
| Gender | 0.009 | 0.059 | 0.154 | 0.878 | 0.009 | 0.007 |
| Highest education | -0.056 | 0.017 | -3.247 | 0.001 | -0.056 | -0.143 |
| Living Conditions | -0.011 | 0.015 | -0.697 | 0.486 | -0.019 | -0.035 |
| **SPQ_total** ~ | |  |  |  |  |  |
| Health before COVID19 | -2.660 | 0.643 | -4.134 | 0.000 | -0.386 | -0.398 |
| Country (residence) | -0.058 | 0.130 | -0.445 | 0.656 | -0.058 | -0.024 |
| Age | -0.007 | 0.003 | -2.659 | 0.008 | -0.007 | -0.109 |
| Gender | -0.202 | 0.104 | -1.949 | 0.051 | -0.202 | -0.089 |
| Highest education | -0.123 | 0.033 | -3.766 | 0.000 | -0.123 | -0.174 |
| Living Conditions | -0.044 | 0.027 | -1.642 | 0.101 | -0.078 | -0.081 |
| **Media** ~ | |  |  |  |  |  |
| Health before COVID19 | -0.334 | 0.187 | -1.790 | 0.074 | -0.048 | -0.105 |
| Country (residence) | -0.053 | 0.052 | -1.026 | 0.305 | -0.053 | -0.048 |
| Age | -0.001 | 0.002 | -0.469 | 0.639 | -0.001 | -0.023 |
| Gender | -0.171 | 0.052 | -3.291 | 0.001 | -0.171 | -0.159 |
| Highest education | -0.039 | 0.016 | -2.447 | 0.014 | -0.039 | -0.115 |
| Living Conditions | -0.021 | 0.014 | -1.423 | 0.155 | -0.036 | -0.079 |
| **Drugs** ~ | |  |  |  |  |  |
| Health before COVID19 | -0.590 | 0.153 | -3.865 | 0.000 | -0.086 | -0.228 |
| Country (residence) | -0.003 | 0.044 | -0.067 | 0.947 | -0.003 | -0.003 |
| Age | 0.001 | 0.001 | 1.162 | 0.245 | 0.001 | 0.050 |
| Gender | 0.015 | 0.042 | 0.370 | 0.712 | 0.015 | 0.018 |
| Highest education | -0.038 | 0.014 | -2.664 | 0.008 | -0.038 | -0.139 |
| Living Conditions | -0.023 | 0.011 | -2.130 | 0.033 | -0.040 | -0.107 |
| **Alcohol** ~ | |  |  |  |  |  |
| Health before COVID19 | 2.950 | 1.115 | 2.647 | 0.008 | 0.428 | 0.189 |
| Country (residence) | 0.050 | 0.251 | 0.198 | 0.843 | 0.050 | 0.009 |
| Age | 0.027 | 0.007 | 3.697 | 0.000 | 0.027 | 0.174 |
| Gender | -0.381 | 0.254 | -1.497 | 0.135 | -0.381 | -0.072 |
| Highest education | 0.258 | 0.082 | 3.151 | 0.002 | 0.258 | 0.157 |
| Living Conditions | 0.092 | 0.063 | 1.457 | 0.145 | 0.162 | 0.072 |
| **Exercise** ~ | |  |  |  |  |  |
| Health before COVID19 | 0.210 | 0.487 | 0.430 | 0.667 | 0.030 | 0.027 |
| Country (residence) | -0.062 | 0.144 | -0.431 | 0.666 | -0.062 | -0.023 |
| Age | 0.001 | 0.004 | 0.318 | 0.751 | 0.001 | 0.017 |
| Gender | -0.049 | 0.131 | -0.375 | 0.707 | -0.049 | -0.019 |
| Highest education | 0.138 | 0.036 | 3.783 | 0.000 | 0.138 | 0.171 |
| Living Conditions | 0.024 | 0.041 | 0.591 | 0.554 | 0.043 | 0.039 |
| **Sleep during week** ~ | |  |  |  |  |  |
| Health before COVID19 | 0.310 | 0.165 | 1.884 | 0.059 | 0.045 | 0.113 |
| Country (residence) | 0.034 | 0.049 | 0.689 | 0.491 | 0.034 | 0.035 |
| Age | 0.000 | 0.001 | 0.069 | 0.945 | 0.000 | 0.003 |
| Gender | 0.045 | 0.047 | 0.950 | 0.342 | 0.045 | 0.048 |
| Highest education | 0.037 | 0.015 | 2.524 | 0.012 | 0.037 | 0.128 |
| Living Conditions | 0.002 | 0.012 | 0.150 | 0.881 | 0.003 | 0.008 |
| **Health before COVI19** ~ | |  |  |  |  |  |
| Country (residence) | 0.044 | 0.021 | 2.097 | 0.036 | 0.305 | 0.126 |
| Age | 0.001 | 0.001 | 1.730 | 0.084 | 0.007 | 0.099 |
| Gender | 0.008 | 0.019 | 0.457 | 0.648 | 0.058 | 0.025 |
| Highest education | -0.008 | 0.006 | -1.333 | 0.182 | -0.055 | -0.075 |
| Living Conditions | -0.003 | 0.006 | -0.521 | 0.602 | -0.035 | -0.035 |
| **depression** ~ | |  |  |  |  |  |
| COVID19 related life concerns (c1) | 0.351 | 0.050 | 7.084 | 0.000 | 0.351 | 0.470 |
| **anxiet**y ~ |  |  |  |  |  |  |
| COVID19 related life concerns (c2) | 0.198 | 0.035 | 5.634 | 0.000 | 0.198 | 0.366 |
| **SPQ_total** ~ | |  |  |  |  |  |
| COVID19 related life concerns (c3) | 0.202 | 0.059 | 3.439 | 0.001 | 0.202 | 0.208 |
| **Media** ~ | |  |  |  |  |  |
| COVID19 related life concerns (a1) | 0.037 | 0.029 | 1.292 | 0.196 | 0.037 | 0.081 |
| **Drugs** ~ | |  |  |  |  |  |
| COVID19 related life concerns (a2) | 0.064 | 0.027 | 2.365 | 0.018 | 0.064 | 0.170 |
| **Alcohol** ~ | |  |  |  |  |  |
| COVID19 related life concerns (a3) | 0.331 | 0.139 | 2.387 | 0.017 | 0.331 | 0.146 |
| **Exercise** ~ | |  |  |  |  |  |
| COVID19 related life concerns (a4) | -0.095 | 0.060 | -1.579 | 0.114 | -0.095 | -0.086 |
| **Sleep during week** ~ | |  |  |  |  |  |
| COVID19 related life concerns (a5) | -0.086 | 0.028 | -3.036 | 0.002 | -0.086 | -0.214 |
| **depression** ~ | |  |  |  |  |  |
| Media (b1) | 0.106 | 0.072 | 1.479 | 0.139 | 0.106 | 0.066 |
| Drugs (b2) | 0.118 | 0.106 | 1.114 | 0.265 | 0.118 | 0.059 |
| Alcohol (b3) | 0.005 | 0.015 | 0.361 | 0.718 | 0.005 | 0.016 |
| Exercise (b4) | 0.006 | 0.029 | 0.212 | 0.832 | 0.006 | 0.009 |
| Sleep during week(b5) | -0.095 | 0.087 | -1.086 | 0.278 | -0.095 | -0.051 |
| **anxiety** ~ |  |  |  |  |  |  |
| Media (b6) | 0.116 | 0.050 | 2.304 | 0.021 | 0.116 | 0.099 |
| Drug (b7) | -0.005 | 0.079 | -0.069 | 0.945 | -0.005 | -0.004 |
| Alcohol (b8) | -0.003 | 0.011 | -0.250 | 0.802 | -0.003 | -0.011 |
| Exercise (b9) | 0.057 | 0.023 | 2.444 | 0.015 | 0.057 | 0.117 |
| Sleep during week (b10) | -0.017 | 0.067 | -0.261 | 0.794 | -0.017 | -0.013 |
| **SPQ_total** ~ | |  |  |  |  |  |
| Media (b11) | 0.023 | 0.092 | 0.255 | 0.799 | 0.023 | 0.011 |
| Drug (b12) | 0.149 | 0.137 | 1.088 | 0.277 | 0.149 | 0.058 |
| Alcohol (b13) | -0.030 | 0.020 | -1.489 | 0.136 | -0.030 | -0.070 |
| Exercise (b14) | 0.035 | 0.043 | 0.805 | 0.421 | 0.035 | 0.040 |
| Sleep during week (b15) | -0.015 | 0.123 | -0.119 | 0.906 | -0.015 | -0.006 |
|  |  |  |  |  |  |  |
| **Covariances:** |  |  |  |  |  |  |
|  | Estimate | Std.Err | z-value | P(>\|z\|) | Std.lv | Std.all |
| .anxiety ~~ |  |  |  |  |  |  |
| .SPQ_total | 0.168 | 0.027 | 6.150 | 0.000 | 0.168 | 0.505 |
| .depression ~~ |  |  |  |  |  |  |
| .SPQ_total | 0.147 | 0.031 | 4.803 | 0.000 | 0.147 | 0.330 |
| .anxiety | 0.107 | 0.020 | 5.278 | 0.000 | 0.107 | 0.473 |
| Living conditions ~~ |  |  |  |  |  |  |
| COVID19 related life concerns | -0.265 | 0.115 | -2.316 | 0.021 | -0.150 | -0.150 |
|  |  |  |  |  |  |  |
| **Variances:** |  |  |  |  |  |  |
|  | Estimate | Std.Err | z-value | P(>\|z\|) | Std.lv | Std.all |
| COVID19 related life concerns | 1.000 |  |  |  | 1.000 | 1.000 |
| .Number rooms | 1.247 | 0.319 | 3.911 | 0.000 | 1.247 | 0.284 |
| .Number people per household | 0.865 | 0.061 | 14.072 | 0.000 | 0.865 | 0.634 |
| .Living Area | 1.427 | 0.102 | 13.939 | 0.000 | 1.427 | 0.792 |
| .Garden_yard | 0.139 | 0.012 | 12.040 | 0.000 | 0.139 | 0.621 |
| .Regular treatment physical illness | 0.091 | 0.010 | 9.293 | 0.000 | 0.091 | 0.811 |
| .Mental health status before COVID19 | 0.341 | 0.086 | 3.966 | 0.000 | 0.341 | 0.339 |
| .Physical health status before COVID19 | 0.502 | 0.053 | 9.530 | 0.000 | 0.502 | 0.599 |
| .Financial Impact due to COVID19 | 0.645 | 0.071 | 9.032 | 0.000 | 0.645 | 0.660 |
| .Concerns about life stability | 0.586 | 0.088 | 6.691 | 0.000 | 0.586 | 0.424 |
| .Restrictions perceived as stressful | 0.968 | 0.091 | 10.643 | 0.000 | 0.968 | 0.639 |
| .depression | 0.303 | 0.032 | 9.540 | 0.000 | 0.303 | 0.542 |
| .anxiety | 0.168 | 0.020 | 8.198 | 0.000 | 0.168 | 0.572 |
| .SPQ_total | 0.657 | 0.059 | 11.038 | 0.000 | 0.657 | 0.698 |
| .Media | 0.200 | 0.010 | 20.910 | 0.000 | 0.200 | 0.936 |
| .Drug | 0.126 | 0.010 | 12.212 | 0.000 | 0.126 | 0.890 |
| .Alcohol | 4.492 | 0.201 | 22.352 | 0.000 | 4.492 | 0.874 |
| .Exercise | 1.183 | 0.079 | 14.905 | 0.000 | 1.183 | 0.959 |
| .Sleep during week | 0.148 | 0.012 | 12.820 | 0.000 | 0.148 | 0.924 |
| Living Conditions | 3.143 | 0.402 | 7.825 | 0.000 | 1.000 | 1.000 |
| .health before COVID19 | 0.020 | 0.007 | 2.853 | 0.004 | 0.965 | 0.965 |
|  |  |  |  |  |  |  |
| **Defined Parameters:** |  |  |  |  |  |  |
|  | Estimate | Std.Err | z-value | P(>\|z\|) | Std.lv | Std.all |
| indirect1 | 0.004 | 0.004 | 0.950 | 0.342 | 0.004 | 0.005 |
| indirect2 | 0.008 | 0.008 | 0.988 | 0.323 | 0.008 | 0.010 |
| indirect3 | 0.002 | 0.005 | 0.339 | 0.735 | 0.002 | 0.002 |
| indirect4 | -0.001 | 0.004 | -0.169 | 0.866 | -0.001 | -0.001 |
| indirect5 | 0.008 | 0.008 | 1.031 | 0.303 | 0.008 | 0.011 |
| indirect6 | 0.004 | 0.004 | 1.111 | 0.266 | 0.004 | 0.008 |
| indirect7 | -0.000 | 0.006 | -0.062 | 0.951 | -0.000 | -0.001 |
| indirect8 | -0.001 | 0.004 | -0.230 | 0.818 | -0.001 | -0.002 |
| indirect9 | -0.005 | 0.005 | -1.174 | 0.240 | -0.005 | -0.010 |
| indirect10 | 0.001 | 0.006 | 0.246 | 0.805 | 0.001 | 0.003 |
| indirect11 | 0.001 | 0.004 | 0.204 | 0.839 | 0.001 | 0.001 |
| indirect12 | 0.010 | 0.010 | 0.967 | 0.334 | 0.010 | 0.010 |
| indirect13 | -0.010 | 0.008 | -1.210 | 0.226 | -0.010 | -0.010 |
| indirect14 | -0.003 | 0.005 | -0.627 | 0.531 | -0.003 | -0.003 |
| indirect15 | 0.001 | 0.011 | 0.115 | 0.909 | 0.001 | 0.001 |
| total1 | 0.355 | 0.050 | 7.120 | 0.000 | 0.355 | 0.475 |
| total2 | 0.359 | 0.049 | 7.276 | 0.000 | 0.359 | 0.480 |
| total3 | 0.353 | 0.048 | 7.290 | 0.000 | 0.353 | 0.472 |
| total4 | 0.351 | 0.049 | 7.150 | 0.000 | 0.351 | 0.469 |
| total5 | 0.359 | 0.047 | 7.589 | 0.000 | 0.359 | 0.481 |
| total6 | 0.203 | 0.036 | 5.665 | 0.000 | 0.203 | 0.374 |
| total7 | 0.198 | 0.034 | 5.759 | 0.000 | 0.198 | 0.366 |
| total8 | 0.198 | 0.035 | 5.716 | 0.000 | 0.198 | 0.365 |
| total9 | 0.193 | 0.035 | 5.537 | 0.000 | 0.193 | 0.356 |
| total10 | 0.200 | 0.033 | 5.989 | 0.000 | 0.200 | 0.369 |
| total11 | 0.203 | 0.059 | 3.453 | 0.001 | 0.203 | 0.209 |
| total12 | 0.211 | 0.058 | 3.655 | 0.000 | 0.211 | 0.218 |
| total13 | 0.192 | 0.058 | 3.301 | 0.001 | 0.192 | 0.198 |
| total14 | 0.198 | 0.058 | 3.412 | 0.001 | 0.198 | 0.205 |
| total15 | 0.203 | 0.056 | 3.611 | 0.000 | 0.203 | 0.209 |
|  |  |  |  |  |  |  |

## ‘Social adversity’ Model – first to forth timepoint

### Social adversity Model – first timepoint

| **Suppl. file 1i. Complete outcome of structural equation with Social adversity as predictor from the first survey timepoint** | | | | | | | | | | | | | | | | | | |  |  |
| --- | --- | --- | --- | --- | --- | --- | --- | --- | --- | --- | --- | --- | --- | --- | --- | --- | --- | --- | --- | --- |
| **Estimator** | **ML** | |  | | |  | | |  | | |  | | |  | | |  |  |  |
| Optimization method | NLMINB | |  | | |  | | |  | | |  | | |  | | |  |  |  |
| Number of free parameters | 108 | |  | | |  | | |  | | |  | | |  | | |  |  |  |
| Number of observations | 480 | |  | | |  | | |  | | |  | | |  | | |  |  |  |
| **Model Test User Model:** |  | |  | | |  | | |  | | |  | | |  | | |  |  |  |
| Test statistic | 492.394 | |  | | |  | | |  | | |  | | |  | | |  |  |  |
| Degrees of freedom | 135 | |  | | |  | | |  | | |  | | |  | | |  |  |  |
| P-value (Chi-square) | 0.000 | |  | | |  | | |  | | |  | | |  | | |  |  |  |
| **Model Test Baseline Model:** |  | |  | | |  | | |  | | |  | | |  | | |  |  |  |
| Test statistic | 2.653.514 | |  | | |  | | |  | | |  | | |  | | |  |  |  |
| Degrees of freedom | 225 | |  | | |  | | |  | | |  | | |  | | |  |  |  |
| P-value | 0.000 | |  | | |  | | |  | | |  | | |  | | |  |  |  |
| **User Model versus Baseline Model:** |  | |  | | |  | | |  | | |  | | |  | | |  |  |  |
| Comparative Fit Index (CFI) | 0.853 | |  | | |  | | |  | | |  | | |  | | |  |  |  |
| Tucker-Lewis Index (TLI) | 0.755 | |  | | |  | | |  | | |  | | |  | | |  |  |  |
| **Loglikelihood and Information Criteria:** |  | |  | | |  | | |  | | |  | | |  | | |  |  |  |
| Loglikelihood user model (H0) | -10089.290 | |  | | |  | | |  | | |  | | |  | | |  |  |  |
| Loglikelihood unrestricted model (H1) | -9843.093 | |  | | |  | | |  | | |  | | |  | | |  |  |  |
| Akaike (AIC) | 20394.580 | |  | | |  | | |  | | |  | | |  | | |  |  |  |
| Bayesian (BIC) | 20845.349 | |  | | |  | | |  | | |  | | |  | | |  |  |  |
| Sample-size adjusted Bayesian (BIC) | 20502.568 | |  | | |  | | |  | | |  | | |  | | |  |  |  |
| **Root Mean Square Error of Approximation:** |  | |  | | |  | | |  | | |  | | |  | | |  |  |  |
| RMSEA | 0.074 | |  | | |  | | |  | | |  | | |  | | |  |  |  |
| 90 Percent confidence interval - lower | 0.067 | |  | | |  | | |  | | |  | | |  | | |  |  |  |
| 90 Percent confidence interval - upper | 0.081 | |  | | |  | | |  | | |  | | |  | | |  |  |  |
| P-value RMSEA <= 0.05 | 0.000 | |  | | |  | | |  | | |  | | |  | | |  |  |  |
| **Standardized Root Mean Square Residual:** |  | |  | | |  | | |  | | |  | | |  | | |  |  |  |
| SRMR | 0.082 | |  | | |  | | |  | | |  | | |  | | |  |  |  |
| **Parameter Estimates:** |  | |  | | |  | | |  | | |  | | |  | | |  |  |  |
| Standard errors | Bootstrap | |  | | |  | | |  | | |  | | |  | | |  |  |  |
| Number of requested bootstrap draws | 1000 | |  | | |  | | |  | | |  | | |  | | |  |  |  |
| Number of successful bootstrap draws | 1000 | |  | | |  | | |  | | |  | | |  | | |  |  |  |
| **Latent Variables:** |  | |  | | |  | | |  | | |  | | |  | | |  |  |  |
|  | Estimate | | Std. err. | | | z-value | | | P(>\|z\|) | | | Std.lv | | | Std.all | | |  |  |  |
| **Living Conditions =~** |  | |  | | |  | | |  | | |  | | |  | | |  |  |  |
| Number_rooms | 1.000 | |  | | |  | | |  | | | 2.182 | | | 0.783 | | |  |  |  |
| Number people per household | 0.236 | | 0.023 | | | 10.085 | | | 0.000 | | | 0.516 | | | 0.649 | | |  |  |  |
| Living Area | 0.323 | | 0.030 | | | 10.671 | | | 0.000 | | | 0.704 | | | 0.521 | | |  |  |  |
| Garden_yard | 0.134 | | 0.014 | | | 9.268 | | | 0.000 | | | 0.293 | | | 0.635 | | |  |  |  |
| **Health before COVID19 =~** | | | |  | | | |  | | |  | | |  | | |  | | | |
| Regular treatment physical illness | 1.000 | |  | | |  | | |  | | | 0.155 | | | 0.457 | | |  |  |  |
| Mental health status before COVID19 | 5.886 | | 1.056 | | | 5.574 | | | 0.000 | | | 0.915 | | | 0.830 | | |  |  |  |
| Physical health status before COVID19 | 3.673 | | 0.695 | | | 5.286 | | | 0.000 | | | 0.571 | | | 0.578 | | |  |  |  |
| **Social adversity =~** | |  | | |  | |  | | |  | | |  | | |  | | | |  |
| Lonely during COVID19 | 0.860 | | 0.061 | | | 14.086 | | | 0.000 | | | 0.860 | | | 0.678 | | |  |  |  |
| Negative Thoughts during COVID19 | 0.813 | | 0.046 | | | 17.639 | | | 0.000 | | | 0.813 | | | 0.754 | | |  |  |  |
| Stressful social relationship changes | 0.765 | | 0.061 | | | 12.575 | | | 0.000 | | | 0.765 | | | 0.566 | | |  |  |  |
|  | |  | | |  | |  | | |  | | |  | | |  | | | |  |
| **Regressions:** |  | |  | | |  | | |  | | |  | | |  | | |  |  |  |
|  | Estimate | | Std.Err | | | z-value | | | P(>\|z\|) | | | Std.lv | | | Std.all | | |  |  |  |
| **depression** ~ | | | |  | | | |  | | |  | | |  | | |  | | | |
| Health before COVID19 | 1.580 | | 0.297 | | | 5.315 | | | 0.000 | | | 0.246 | | | 0.368 | | |  |  |  |
| Country (residence) | -0.304 | | 0.057 | | | -5.366 | | | 0.000 | | | -0.304 | | | -0.205 | | |  |  |  |
| Age | 0.020 | | 0.023 | | | 0.887 | | | 0.375 | | | 0.020 | | | 0.030 | | |  |  |  |
| Gender | 0.063 | | 0.048 | | | 1.298 | | | 0.194 | | | 0.063 | | | 0.041 | | |  |  |  |
| Highest education | -0.051 | | 0.018 | | | -2.777 | | | 0.005 | | | -0.051 | | | -0.091 | | |  |  |  |
| Living Conditions | 0.023 | | 0.013 | | | 1.687 | | | 0.092 | | | 0.049 | | | 0.074 | | |  |  |  |
| **anxiety** ~ |  | |  | | |  | | |  | | |  | | |  | | |  |  |  |
| Health before COVID19 | 1.198 | | 0.252 | | | 4.753 | | | 0.000 | | | 0.186 | | | 0.336 | | |  |  |  |
| Country (residence) | -0.260 | | 0.059 | | | -4.417 | | | 0.000 | | | -0.260 | | | -0.211 | | |  |  |  |
| Age | -0.045 | | 0.021 | | | -2.089 | | | 0.037 | | | -0.045 | | | -0.081 | | |  |  |  |
| Gender | 0.024 | | 0.044 | | | 0.537 | | | 0.591 | | | 0.024 | | | 0.019 | | |  |  |  |
| Highest education | -0.005 | | 0.019 | | | -0.269 | | | 0.788 | | | -0.005 | | | -0.011 | | |  |  |  |
| Living Conditions | 0.017 | | 0.014 | | | 1.159 | | | 0.246 | | | 0.036 | | | 0.065 | | |  |  |  |
| **SPQ_total** ~ | | | |  | | | |  | | |  | | |  | | |  | | | |
| Health before COVID19 | 2.472 | | 0.506 | | | 4.889 | | | 0.000 | | | 0.384 | | | 0.403 | | |  |  |  |
| Country (residence) | 0.012 | | 0.109 | | | 0.112 | | | 0.911 | | | 0.012 | | | 0.006 | | |  |  |  |
| Age | -0.070 | | 0.047 | | | -1.503 | | | 0.133 | | | -0.070 | | | -0.074 | | |  |  |  |
| Gender | -0.149 | | 0.090 | | | -1.658 | | | 0.097 | | | -0.149 | | | -0.069 | | |  |  |  |
| Highest education | -0.069 | | 0.039 | | | -1.774 | | | 0.076 | | | -0.069 | | | -0.087 | | |  |  |  |
| Living Conditions | -0.014 | | 0.025 | | | -0.545 | | | 0.585 | | | -0.030 | | | -0.031 | | |  |  |  |
| **Media** ~ | | | |  | | | |  | | |  | | |  | | |  | | | |
| Health before COVID19 | 0.043 | | 0.196 | | | 0.219 | | | 0.827 | | | 0.007 | | | 0.014 | | |  |  |  |
| Country (residence) | -0.171 | | 0.050 | | | -3.389 | | | 0.001 | | | -0.171 | | | -0.166 | | |  |  |  |
| Age | -0.065 | | 0.021 | | | -3.028 | | | 0.002 | | | -0.065 | | | -0.139 | | |  |  |  |
| Gender | -0.001 | | 0.046 | | | -0.015 | | | 0.988 | | | -0.001 | | | -0.001 | | |  |  |  |
| Highest education | -0.049 | | 0.017 | | | -2.872 | | | 0.004 | | | -0.049 | | | -0.127 | | |  |  |  |
| Living Conditions | -0.019 | | 0.012 | | | -1.558 | | | 0.119 | | | -0.041 | | | -0.088 | | |  |  |  |
| **Drugs** ~ | | | |  | | | |  | | |  | | |  | | |  | | | |
| Health before COVID19 | 0.454 | | 0.153 | | | 2.958 | | | 0.003 | | | 0.071 | | | 0.213 | | |  |  |  |
| Country (residence) | 0.010 | | 0.036 | | | 0.287 | | | 0.774 | | | 0.010 | | | 0.014 | | |  |  |  |
| Age | 0.032 | | 0.016 | | | 2.029 | | | 0.042 | | | 0.032 | | | 0.095 | | |  |  |  |
| Gender | -0.003 | | 0.033 | | | -0.088 | | | 0.930 | | | -0.003 | | | -0.004 | | |  |  |  |
| Highest education | -0.008 | | 0.013 | | | -0.644 | | | 0.519 | | | -0.008 | | | -0.029 | | |  |  |  |
| Living Conditions | -0.002 | | 0.008 | | | -0.238 | | | 0.812 | | | -0.004 | | | -0.012 | | |  |  |  |
| **Alcohol** ~ | | | |  | | | |  | | |  | | |  | | |  | | | |
| Health before COVID19 | -2.456 | | 0.953 | | | -2.578 | | | 0.010 | | | -0.382 | | | -0.167 | | |  |  |  |
| Country (residence) | -0.512 | | 0.229 | | | -2.238 | | | 0.025 | | | -0.512 | | | -0.101 | | |  |  |  |
| Age | 0.507 | | 0.102 | | | 4.991 | | | 0.000 | | | 0.507 | | | 0.221 | | |  |  |  |
| Gender | -0.470 | | 0.229 | | | -2.047 | | | 0.041 | | | -0.470 | | | -0.091 | | |  |  |  |
| Highest education | 0.038 | | 0.088 | | | 0.432 | | | 0.666 | | | 0.038 | | | 0.020 | | |  |  |  |
| Living Conditions | 0.068 | | 0.056 | | | 1.200 | | | 0.230 | | | 0.148 | | | 0.065 | | |  |  |  |
| **Exercise** ~ | | | |  | | | |  | | |  | | |  | | |  | | | |
| Health before COVID19 | -1.301 | | 0.571 | | | -2.277 | | | 0.023 | | | -0.202 | | | -0.162 | | |  |  |  |
| Country (residence) | -0.523 | | 0.138 | | | -3.793 | | | 0.000 | | | -0.523 | | | -0.189 | | |  |  |  |
| Age | 0.175 | | 0.059 | | | 2.988 | | | 0.003 | | | 0.175 | | | 0.140 | | |  |  |  |
| Gender | -0.063 | | 0.129 | | | -0.490 | | | 0.624 | | | -0.063 | | | -0.022 | | |  |  |  |
| Highest education | 0.035 | | 0.046 | | | 0.774 | | | 0.439 | | | 0.035 | | | 0.034 | | |  |  |  |
| Living Conditions | -0.006 | | 0.030 | | | -0.191 | | | 0.849 | | | -0.013 | | | -0.010 | | |  |  |  |
| **Sleep during week** ~ | | | |  | | | |  | | |  | | |  | | |  | | | |
| Health before COVID19 | 0.153 | | 0.254 | | | 0.600 | | | 0.548 | | | 0.024 | | | 0.037 | | |  |  |  |
| Country (residence) | -0.089 | | 0.070 | | | -1.283 | | | 0.199 | | | -0.089 | | | -0.063 | | |  |  |  |
| Age | -0.093 | | 0.031 | | | -3.025 | | | 0.002 | | | -0.093 | | | -0.144 | | |  |  |  |
| Gender | 0.094 | | 0.060 | | | 1.550 | | | 0.121 | | | 0.094 | | | 0.065 | | |  |  |  |
| Highest education | -0.036 | | 0.024 | | | -1.508 | | | 0.132 | | | -0.036 | | | -0.068 | | |  |  |  |
| Living Conditions | -0.023 | | 0.015 | | | -1.513 | | | 0.130 | | | -0.051 | | | -0.080 | | |  |  |  |
| **Health before COVI19** ~ | | | |  | | | |  | | |  | | |  | | |  | | | |
| Country (residence) | -0.058 | | 0.021 | | | -2.732 | | | 0.006 | | | -0.374 | | | -0.169 | | |  |  |  |
| Age | -0.024 | | 0.010 | | | -2.485 | | | 0.013 | | | -0.154 | | | -0.154 | | |  |  |  |
| Gender | 0.043 | | 0.020 | | | 2.161 | | | 0.031 | | | 0.275 | | | 0.122 | | |  |  |  |
| Highest education | -0.019 | | 0.008 | | | -2.500 | | | 0.012 | | | -0.124 | | | -0.148 | | |  |  |  |
| Living Conditions | -0.008 | | 0.005 | | | -1.453 | | | 0.146 | | | -0.110 | | | -0.110 | | |  |  |  |
| **depression** ~ | | | |  | | | |  | | |  | | |  | | |  | | | |
| Social adversity (c1) | 0.474 | | 0.035 | | | 13.362 | | | 0.000 | | | 0.474 | | | 0.710 | | |  |  |  |
| **anxiet**y ~ |  | |  | | |  | | |  | | |  | | |  | | |  |  |  |
| Social adversity (c2) | 0.253 | | 0.033 | | | 7.582 | | | 0.000 | | | 0.253 | | | 0.456 | | |  |  |  |
| **SPQ_total** ~ | | | |  | | | |  | | |  | | |  | | |  | | | |
| Social adversity (c3) | 0.118 | | 0.058 | | | 2.021 | | | 0.043 | | | 0.118 | | | 0.123 | | |  |  |  |
| **Media** ~ | | | |  | | | |  | | |  | | |  | | |  | | | |
| Social adversity (a1) | 0.050 | | 0.027 | | | 1.866 | | | 0.062 | | | 0.050 | | | 0.108 | | |  |  |  |
| **Drugs** ~ | | | |  | | | |  | | |  | | |  | | |  | | | |
| Social adversity (a2) | 0.065 | | 0.020 | | | 3.223 | | | 0.001 | | | 0.065 | | | 0.196 | | |  |  |  |
| **Alcohol** ~ | | | |  | | | |  | | |  | | |  | | |  | | | |
| Social adversity (a3) | 0.497 | | 0.127 | | | 3.905 | | | 0.000 | | | 0.497 | | | 0.218 | | |  |  |  |
| **Exercise** ~ | | | |  | | | |  | | |  | | |  | | |  | | | |
| Social adversity (a4) | 0.029 | | 0.078 | | | 0.366 | | | 0.715 | | | 0.029 | | | 0.023 | | |  |  |  |
| **Sleep during week** ~ | | | |  | | | |  | | |  | | |  | | |  | | | |
| Social adversity (a5) | -0.158 | | 0.038 | | | -4.196 | | | 0.000 | | | -0.158 | | | -0.246 | | |  |  |  |
| **depression** ~ | | | |  | | | |  | | |  | | |  | | |  | | | |
| Media (b1) | 0.040 | | 0.050 | | | 0.789 | | | 0.430 | | | 0.040 | | | 0.028 | | |  |  |  |
| Drugs (b2) | -0.031 | | 0.083 | | | -0.375 | | | 0.708 | | | -0.031 | | | -0.015 | | |  |  |  |
| Alcohol (b3) | 0.005 | | 0.011 | | | 0.436 | | | 0.663 | | | 0.005 | | | 0.016 | | |  |  |  |
| Exercise (b4) | -0.029 | | 0.018 | | | -1.586 | | | 0.113 | | | -0.029 | | | -0.055 | | |  |  |  |
| Sleep during week(b5) | -0.034 | | 0.038 | | | -0.894 | | | 0.371 | | | -0.034 | | | -0.033 | | |  |  |  |
| **anxiety** ~ |  | |  | | |  | | |  | | |  | | |  | | |  |  |  |
| Media (b6) | 0.063 | | 0.052 | | | 1.211 | | | 0.226 | | | 0.063 | | | 0.053 | | |  |  |  |
| Drug (b7) | -0.009 | | 0.085 | | | -0.106 | | | 0.915 | | | -0.009 | | | -0.005 | | |  |  |  |
| Alcohol (b8) | -0.007 | | 0.010 | | | -0.694 | | | 0.487 | | | -0.007 | | | -0.030 | | |  |  |  |
| Exercise (b9) | 0.001 | | 0.020 | | | 0.061 | | | 0.951 | | | 0.001 | | | 0.003 | | |  |  |  |
| Sleep during week (b10) | -0.024 | | 0.038 | | | -0.637 | | | 0.524 | | | -0.024 | | | -0.028 | | |  |  |  |
| **SPQ_total** ~ | | | |  | | | |  | | |  | | |  | | |  | | | |
| Media (b11) | 0.213 | | 0.093 | | | 2.294 | | | 0.022 | | | 0.213 | | | 0.104 | | |  |  |  |
| Drug (b12) | 0.133 | | 0.142 | | | 0.931 | | | 0.352 | | | 0.133 | | | 0.046 | | |  |  |  |
| Alcohol (b13) | 0.002 | | 0.020 | | | 0.125 | | | 0.901 | | | 0.002 | | | 0.006 | | |  |  |  |
| Exercise (b14) | -0.044 | | 0.034 | | | -1.277 | | | 0.202 | | | -0.044 | | | -0.058 | | |  |  |  |
| Sleep during week (b15) | -0.085 | | 0.069 | | | -1.235 | | | 0.217 | | | -0.085 | | | -0.057 | | |  |  |  |
|  | |  | | |  | |  | | |  | | |  | | |  | | | |  |
| **Covariances:** |  | |  | | |  | | |  | | |  | | |  | | |  |  |  |
|  | Estimate | | Std.Err | | | z-value | | | P(>\|z\|) | | | Std.lv | | | Std.all | | |  |  |  |
| .anxiety ~~ |  | |  | | |  | | |  | | |  | | |  | | |  |  |  |
| .SPQ_total | 0.125 | | 0.023 | | | 5.436 | | | 0.000 | | | 0.125 | | | 0.366 | | |  |  |  |
| .depression ~~ |  | |  | | |  | | |  | | |  | | |  | | |  |  |  |
| .SPQ_total | 0.034 | | 0.021 | | | 1.592 | | | 0.111 | | | 0.034 | | | 0.121 | | |  |  |  |
| .anxiety | 0.036 | | 0.012 | | | 3.070 | | | 0.002 | | | 0.036 | | | 0.254 | | |  |  |  |
| Living conditions~~ |  | |  | | |  | | |  | | |  | | |  | | |  |  |  |
| Social adversity | -0.355 | | 0.146 | | | -2.427 | | | 0.015 | | | -0.162 | | | -0.162 | | |  |  |  |
|  |  | |  | | |  | | |  | | |  | | |  | | |  |  |  |
| **Variances:** |  | |  | | |  | | |  | | |  | | |  | | |  |  |  |
|  | Estimate | | Std.Err | | | z-value | | | P(>\|z\|) | | | Std.lv | | | Std.all | | |  |  |  |
| Social adversity | 1.000 | |  | | |  | | |  | | | 1.000 | | | 1.000 | | |  |  |  |
| .Number rooms | 3.014 | | 0.473 | | | 6.376 | | | 0.000 | | | 3.014 | | | 0.388 | | |  |  |  |
| .Number people per household | 0.365 | | 0.028 | | | 13.020 | | | 0.000 | | | 0.365 | | | 0.579 | | |  |  |  |
| .Living Area | 1.330 | | 0.097 | | | 13.752 | | | 0.000 | | | 1.330 | | | 0.728 | | |  |  |  |
| .Garden_yard | 0.127 | | 0.010 | | | 12.671 | | | 0.000 | | | 0.127 | | | 0.596 | | |  |  |  |
| .Regular treatment physical illness | 0.092 | | 0.009 | | | 10.016 | | | 0.000 | | | 0.092 | | | 0.792 | | |  |  |  |
| .Mental health status before COVID19 | 0.378 | | 0.103 | | | 3.657 | | | 0.000 | | | 0.378 | | | 0.311 | | |  |  |  |
| .Physical health status before COVID19 | 0.651 | | 0.053 | | | 12.182 | | | 0.000 | | | 0.651 | | | 0.666 | | |  |  |  |
| .Lonely during COVID19 | 0.871 | | 0.076 | | | 11.395 | | | 0.000 | | | 0.871 | | | 0.541 | | |  |  |  |
| .Negative Thoughts during COVID19 | 0.502 | | 0.057 | | | 8.801 | | | 0.000 | | | 0.502 | | | 0.432 | | |  |  |  |
| .stressful social relationships changes | 1.242 | | 0.090 | | | 13.764 | | | 0.000 | | | 1.242 | | | 0.680 | | |  |  |  |
| .depression | 0.118 | | 0.015 | | | 7.746 | | | 0.000 | | | 0.118 | | | 0.264 | | |  |  |  |
| .anxiety | 0.173 | | 0.018 | | | 9.867 | | | 0.000 | | | 0.173 | | | 0.566 | | |  |  |  |
| .SPQ_total | 0.667 | | 0.060 | | | 11.204 | | | 0.000 | | | 0.667 | | | 0.733 | | |  |  |  |
| .Media | 0.194 | | 0.010 | | | 20.310 | | | 0.000 | | | 0.194 | | | 0.903 | | |  |  |  |
| .Drug | 0.099 | | 0.010 | | | 10.313 | | | 0.000 | | | 0.099 | | | 0.911 | | |  |  |  |
| .Alcohol | 4.405 | | 0.205 | | | 21.526 | | | 0.000 | | | 4.405 | | | 0.846 | | |  |  |  |
| .Exercise | 1.441 | | 0.074 | | | 19.587 | | | 0.000 | | | 1.441 | | | 0.924 | | |  |  |  |
| .Sleep during week | 0.367 | | 0.026 | | | 14.023 | | | 0.000 | | | 0.367 | | | 0.894 | | |  |  |  |
| Living Conditons | 4.763 | | 0.578 | | | 8.235 | | | 0.000 | | | 1.000 | | | 1.000 | | |  |  |  |
| .health before COVID19 | 0.021 | | 0.006 | | | 3.460 | | | 0.001 | | | 0.885 | | | 0.885 | | |  |  |  |
|  |  | |  | | |  | | |  | | |  | | |  | | |  |  |  |
| **Defined Parameters:** |  | |  | | |  | | |  | | |  | | |  | | |  |  |  |
|  | Estimate | | Std.Err | | | z-value | | | P(>\|z\|) | | | Std.lv | | | Std.all | | |  |  |  |
| indirect1 | 0.002 | | 0.003 | | | 0.692 | | | 0.489 | | | 0.002 | | | 0.003 | | |  |  |  |
| indirect2 | -0.002 | | 0.006 | | | -0.345 | | | 0.730 | | | -0.002 | | | -0.003 | | |  |  |  |
| indirect3 | 0.002 | | 0.006 | | | 0.430 | | | 0.667 | | | 0.002 | | | 0.004 | | |  |  |  |
| indirect4 | -0.001 | | 0.003 | | | -0.304 | | | 0.761 | | | -0.001 | | | -0.001 | | |  |  |  |
| indirect5 | 0.005 | | 0.006 | | | 0.870 | | | 0.384 | | | 0.005 | | | 0.008 | | |  |  |  |
| indirect6 | 0.003 | | 0.003 | | | 0.954 | | | 0.340 | | | 0.003 | | | 0.006 | | |  |  |  |
| indirect7 | -0.001 | | 0.006 | | | -0.103 | | | 0.918 | | | -0.001 | | | -0.001 | | |  |  |  |
| indirect8 | -0.004 | | 0.005 | | | -0.655 | | | 0.513 | | | -0.004 | | | -0.006 | | |  |  |  |
| indirect9 | 0.000 | | 0.002 | | | 0.022 | | | 0.982 | | | 0.000 | | | 0.000 | | |  |  |  |
| indirect10 | 0.004 | | 0.006 | | | 0.614 | | | 0.539 | | | 0.004 | | | 0.007 | | |  |  |  |
| indirect11 | 0.011 | | 0.008 | | | 1.418 | | | 0.156 | | | 0.011 | | | 0.011 | | |  |  |  |
| indirect12 | 0.009 | | 0.010 | | | 0.873 | | | 0.383 | | | 0.009 | | | 0.009 | | |  |  |  |
| indirect13 | 0.001 | | 0.010 | | | 0.122 | | | 0.903 | | | 0.001 | | | 0.001 | | |  |  |  |
| indirect14 | -0.001 | | 0.005 | | | -0.273 | | | 0.785 | | | -0.001 | | | -0.001 | | |  |  |  |
| indirect15 | 0.013 | | 0.012 | | | 1.133 | | | 0.257 | | | 0.013 | | | 0.014 | | |  |  |  |
| total1 | 0.476 | | 0.035 | | | 13.436 | | | 0.000 | | | 0.476 | | | 0.713 | | |  |  |  |
| total2 | 0.472 | | 0.036 | | | 13.291 | | | 0.000 | | | 0.472 | | | 0.707 | | |  |  |  |
| total3 | 0.477 | | 0.034 | | | 13.963 | | | 0.000 | | | 0.477 | | | 0.714 | | |  |  |  |
| total4 | 0.473 | | 0.036 | | | 13.290 | | | 0.000 | | | 0.473 | | | 0.709 | | |  |  |  |
| total5 | 0.480 | | 0.036 | | | 13.433 | | | 0.000 | | | 0.480 | | | 0.718 | | |  |  |  |
| total6 | 0.256 | | 0.034 | | | 7.632 | | | 0.000 | | | 0.256 | | | 0.462 | | |  |  |  |
| total7 | 0.252 | | 0.033 | | | 7.642 | | | 0.000 | | | 0.252 | | | 0.455 | | |  |  |  |
| total8 | 0.249 | | 0.033 | | | 7.517 | | | 0.000 | | | 0.249 | | | 0.450 | | |  |  |  |
| total9 | 0.253 | | 0.033 | | | 7.587 | | | 0.000 | | | 0.253 | | | 0.456 | | |  |  |  |
| total10 | 0.257 | | 0.034 | | | 7.568 | | | 0.000 | | | 0.257 | | | 0.463 | | |  |  |  |
| total11 | 0.128 | | 0.058 | | | 2.207 | | | 0.027 | | | 0.128 | | | 0.134 | | |  |  |  |
| total12 | 0.126 | | 0.058 | | | 2.188 | | | 0.029 | | | 0.126 | | | 0.132 | | |  |  |  |
| total13 | 0.119 | | 0.057 | | | 2.076 | | | 0.038 | | | 0.119 | | | 0.125 | | |  |  |  |
| total14 | 0.116 | | 0.059 | | | 1.986 | | | 0.047 | | | 0.116 | | | 0.122 | | |  |  |  |
| total15 | 0.131 | | 0.056 | | | 2.331 | | | 0.020 | | | 0.131 | | | 0.137 | | |  |  |  |
|  | |  | | |  | |  | | |  | | |  | | |  | | | |  |

### Social adversity Model – second timepoint

| **Suppl. file 1j. Complete outcome of structural equation with Social adversity as predictor from second survey timepoint.** | | | | | | | | | | | | |  |
| --- | --- | --- | --- | --- | --- | --- | --- | --- | --- | --- | --- | --- | --- |
| **Estimator** | **ML** | |  | |  | |  | |  | |  | | |
| Optimization method | NLMINB | |  | |  | |  | |  | |  | | |
| Number of free parameters | 108 | |  | |  | |  | |  | |  | | |
|  | Used | | Total | |  | |  | |  | |  | | |
| Number of observations | 423 | | 464 | |  | |  | |  | |  | | |
| **Model Test User Model:** |  | |  | |  | |  | |  | |  | | |
| Test statistic | 481.871 | |  | |  | |  | |  | |  | | |
| Degrees of freedom | 135 | |  | |  | |  | |  | |  | | |
| P-value (Chi-square) | 0.000 | |  | |  | |  | |  | |  | | |
| **Model Test Baseline Model:** |  | |  | |  | |  | |  | |  | | |
| Test statistic | 2.645.369 | |  | |  | |  | |  | |  | | |
| Degrees of freedom | 225 | |  | |  | |  | |  | |  | | |
| P-value | 0.000 | |  | |  | |  | |  | |  | | |
| **User Model versus Baseline Model:** |  | |  | |  | |  | |  | |  | | |
| Comparative Fit Index (CFI) | 0.857 | |  | |  | |  | |  | |  | | |
| Tucker-Lewis Index (TLI) | 0.761 | |  | |  | |  | |  | |  | | |
| **Loglikelihood and Information Criteria:** |  | |  | |  | |  | |  | |  | | |
| Loglikelihood user model (H0) | -8297.995 | |  | |  | |  | |  | |  | | |
| Loglikelihood unrestricted model (H1) | -8057.059 | |  | |  | |  | |  | |  | | |
| Akaike (AIC) | 16811.989 | |  | |  | |  | |  | |  | | |
| Bayesian (BIC) | 17249.105 | |  | |  | |  | |  | |  | | |
| Sample-size adjusted Bayesian (BIC) | 16906.385 | |  | |  | |  | |  | |  | | |
| **Root Mean Square Error of Approximation:** |  | |  | |  | |  | |  | |  | | |
| RMSEA | 0.078 | |  | |  | |  | |  | |  | | |
| 90 Percent confidence interval - lower | 0.070 | |  | |  | |  | |  | |  | | |
| 90 Percent confidence interval - upper | 0.086 | |  | |  | |  | |  | |  | | |
| P-value RMSEA <= 0.05 | 0.000 | |  | |  | |  | |  | |  | | |
| **Standardized Root Mean Square Residual:** |  | |  | |  | |  | |  | |  | | |
| SRMR | 0.084 | |  | |  | |  | |  | |  | | |
| **Parameter Estimates:** |  | |  | |  | |  | |  | |  | | |
| Standard errors | Bootstrap | |  | |  | |  | |  | |  | | |
| Number of requested bootstrap draws | 1000 | |  | |  | |  | |  | |  | | |
| Number of successful bootstrap draws | 1000 | |  | |  | |  | |  | |  | | |
| **Latent Variables:** |  | |  | |  | |  | |  | |  | | |
|  | Estimate | | Std.Err | | z-value | | P(>\|z\|) | | Std.lv | | Std.all | | |
| **Living Conditions =~** |  | |  | |  | |  | |  | |  | | |
| Number_rooms | 1.000 | |  | |  | |  | | 2.028 | | 0.920 | | |
| Number people per household | 0.400 | | 0.035 | | 11.569 | | 0.000 | | 0.812 | | 0.642 | | |
| Living Area | 0.336 | | 0.041 | | 8.242 | | 0.000 | | 0.682 | | 0.499 | | |
| Garden_yard | 0.132 | | 0.017 | | 7.974 | | 0.000 | | 0.267 | | 0.568 | | |
| **Health before COVID19 =~** | |  | |  | |  | |  | |  | |  |  |
| Regular treatment physical illness | 1.000 | |  | |  | |  | | 0.163 | | 0.504 | | |
| Mental health status before COVID19 | -5.060 | | 1.315 | | -3.849 | | 0.000 | | -0.825 | | -0.812 | | |
| Physical health status before COVID19 | -3.036 | | 0.675 | | -4.501 | | 0.000 | | -0.495 | | -0.541 | | |
| **Social adversity =~** |  | |  | |  | |  | |  | |  | | |
| Lonely during COVID19 | 0.758 | | 0.065 | | 11.705 | | 0.000 | | 0.758 | | 0.636 | | |
| Negative Thoughts during COVID19 | 0.826 | | 0.049 | | 16.965 | | 0.000 | | 0.826 | | 0.778 | | |
| Stressful social relationship changes | 0.742 | | 0.069 | | 10.697 | | 0.000 | | 0.742 | | 0.616 | | |
|  |  | |  | |  | |  | |  | |  | | |
| **Regressions:** |  | |  | |  | |  | |  | |  | | |
|  | Estimate | | Std.Err | | z-value | | P(>\|z\|) | | Std.lv | | Std.all | | |
| **depression** ~ |  | |  | |  | |  | |  | |  | | |
| Health before COVID19 | -0.944 | | 0.284 | | -3.324 | | 0.001 | | -0.154 | | -0.234 | | |
| Country (residence) | -0.357 | | 0.070 | | -5.092 | | 0.000 | | -0.357 | | -0.229 | | |
| Age | -0.002 | | 0.001 | | -1.114 | | 0.265 | | -0.002 | | -0.039 | | |
| Gender | -0.025 | | 0.053 | | -0.466 | | 0.641 | | -0.025 | | -0.017 | | |
| Highest education | -0.010 | | 0.019 | | -0.519 | | 0.604 | | -0.010 | | -0.019 | | |
| Living Conditions | -0.024 | | 0.014 | | -1.654 | | 0.098 | | -0.048 | | -0.074 | | |
| **anxiety** ~ |  | |  | |  | |  | |  | |  | | |
| Health before COVID19 | -0.932 | | 0.227 | | -4.111 | | 0.000 | | -0.152 | | -0.285 | | |
| Country (residence) | -0.209 | | 0.060 | | -3.474 | | 0.001 | | -0.209 | | -0.165 | | |
| Age | -0.006 | | 0.001 | | -4.865 | | 0.000 | | -0.006 | | -0.165 | | |
| Gender | 0.012 | | 0.048 | | 0.248 | | 0.804 | | 0.012 | | 0.010 | | |
| Highest education | -0.052 | | 0.019 | | -2.767 | | 0.006 | | -0.052 | | -0.121 | | |
| Living Conditions | -0.001 | | 0.013 | | -0.074 | | 0.941 | | -0.002 | | -0.004 | | |
| **SPQ_total** ~ |  | |  | |  | |  | |  | |  | | |
| Health before COVID19 | -2.295 | | 0.473 | | -4.851 | | 0.000 | | -0.374 | | -0.395 | | |
| Country (residence) | -0.064 | | 0.118 | | -0.546 | | 0.585 | | -0.064 | | -0.029 | | |
| Age | -0.005 | | 0.002 | | -2.066 | | 0.039 | | -0.005 | | -0.083 | | |
| Gender | -0.226 | | 0.100 | | -2.268 | | 0.023 | | -0.226 | | -0.106 | | |
| Highest education | -0.129 | | 0.034 | | -3.758 | | 0.000 | | -0.129 | | -0.168 | | |
| Living Conditions | 0.006 | | 0.023 | | 0.268 | | 0.789 | | 0.013 | | 0.013 | | |
| **Media** ~ |  | |  | |  | |  | |  | |  | | |
| Health before COVID19 | -0.181 | | 0.175 | | -1.035 | | 0.301 | | -0.030 | | -0.072 | | |
| Country (residence) | -0.131 | | 0.047 | | -2.764 | | 0.006 | | -0.131 | | -0.133 | | |
| Age | -0.003 | | 0.001 | | -2.249 | | 0.025 | | -0.003 | | -0.117 | | |
| Gender | -0.036 | | 0.043 | | -0.832 | | 0.405 | | -0.036 | | -0.039 | | |
| Highest education | -0.080 | | 0.016 | | -5.008 | | 0.000 | | -0.080 | | -0.238 | | |
| Living Conditions | -0.008 | | 0.011 | | -0.756 | | 0.450 | | -0.017 | | -0.041 | | |
| **Drugs** ~ |  | |  | |  | |  | |  | |  | | |
| Health before COVID19 | -0.164 | | 0.186 | | -0.885 | | 0.376 | | -0.027 | | -0.079 | | |
| Country (residence) | -0.098 | | 0.045 | | -2.164 | | 0.031 | | -0.098 | | -0.122 | | |
| Age | -0.000 | | 0.001 | | -0.224 | | 0.823 | | -0.000 | | -0.010 | | |
| Gender | 0.026 | | 0.035 | | 0.750 | | 0.453 | | 0.026 | | 0.035 | | |
| Highest education | -0.035 | | 0.015 | | -2.384 | | 0.017 | | -0.035 | | -0.129 | | |
| Living Conditions | -0.021 | | 0.008 | | -2.630 | | 0.009 | | -0.044 | | -0.129 | | |
| **Alcohol** ~ |  | |  | |  | |  | |  | |  | | |
| Health before COVID19 | 1.037 | | 1.001 | | 1.036 | | 0.300 | | 0.169 | | 0.079 | | |
| Country (residence) | -0.408 | | 0.245 | | -1.668 | | 0.095 | | -0.408 | | -0.080 | | |
| Age | 0.021 | | 0.007 | | 2.995 | | 0.003 | | 0.021 | | 0.152 | | |
| Gender | -0.448 | | 0.246 | | -1.817 | | 0.069 | | -0.448 | | -0.092 | | |
| Highest education | 0.174 | | 0.088 | | 1.979 | | 0.048 | | 0.174 | | 0.100 | | |
| Living Conditions | 0.064 | | 0.056 | | 1.144 | | 0.253 | | 0.130 | | 0.061 | | |
| **Exercise** ~ | |  | |  | |  | |  | |  | |  |  |
| Health before COVID19 | 0.851 | | 0.518 | | 1.642 | | 0.101 | | 0.139 | | 0.134 | | |
| Country (residence) | -0.254 | | 0.135 | | -1.879 | | 0.060 | | -0.254 | | -0.103 | | |
| Age | -0.001 | | 0.003 | | -0.188 | | 0.851 | | -0.001 | | -0.010 | | |
| Gender | -0.174 | | 0.125 | | -1.392 | | 0.164 | | -0.174 | | -0.074 | | |
| Highest education | 0.059 | | 0.041 | | 1.449 | | 0.147 | | 0.059 | | 0.070 | | |
| Living Conditions | -0.004 | | 0.026 | | -0.169 | | 0.866 | | -0.009 | | -0.009 | | |
| **Sleep during week** ~ | |  | |  | |  | |  | |  | |  |  |
| Health before COVID19 | 0.216 | | 0.151 | | 1.425 | | 0.154 | | 0.035 | | 0.102 | | |
| Country (residence) | 0.071 | | 0.042 | | 1.697 | | 0.090 | | 0.071 | | 0.086 | | |
| Age | -0.002 | | 0.001 | | -2.154 | | 0.031 | | -0.002 | | -0.100 | | |
| Gender | 0.056 | | 0.037 | | 1.507 | | 0.132 | | 0.056 | | 0.072 | | |
| Highest education | 0.024 | | 0.016 | | 1.505 | | 0.132 | | 0.024 | | 0.087 | | |
| Living Conditions | -0.000 | | 0.009 | | -0.031 | | 0.975 | | -0.001 | | -0.002 | | |
| **Health before COVI19** ~ | |  | |  | |  | |  | |  | |  |  |
| Country (residence) | 0.087 | | 0.030 | | 2.873 | | 0.004 | | 0.532 | | 0.224 | | |
| Age | 0.001 | | 0.001 | | 1.402 | | 0.161 | | 0.005 | | 0.084 | | |
| Gender | 0.020 | | 0.020 | | 1.025 | | 0.305 | | 0.123 | | 0.054 | | |
| Highest education | 0.015 | | 0.010 | | 1.409 | | 0.159 | | 0.089 | | 0.110 | | |
| Living Conditions | 0.017 | | 0.007 | | 2.436 | | 0.015 | | 0.210 | | 0.210 | | |
| **depression** ~ |  | |  | |  | |  | |  | |  | | |
| Social adversity (c1) | 0.463 | | 0.040 | | 11.699 | | 0.000 | | 0.463 | | 0.705 | | |
| **anxiet**y ~ |  | |  | |  | |  | |  | |  | | |
| Social adversity (c2) | 0.268 | | 0.041 | | 6.567 | | 0.000 | | 0.268 | | 0.503 | | |
| **SPQ_total** ~ |  | |  | |  | |  | |  | |  | | |
| Social adversity (c3) | 0.237 | | 0.068 | | 3.476 | | 0.001 | | 0.237 | | 0.250 | | |
| **Media** ~ |  | |  | |  | |  | |  | |  | | |
| Social adversity (a1) | 0.042 | | 0.027 | | 1.546 | | 0.122 | | 0.042 | | 0.101 | | |
| **Drugs** ~ |  | |  | |  | |  | |  | |  | | |
| Social adversity (a2) | 0.049 | | 0.028 | | 1.770 | | 0.077 | | 0.049 | | 0.144 | | |
| **Alcohol** ~ |  | |  | |  | |  | |  | |  | | |
| Social adversity (a3) | -0.084 | | 0.148 | | -0.568 | | 0.570 | | -0.084 | | -0.039 | | |
| **Exercise** ~ | |  | |  | |  | |  | |  | |  |  |
| Social adversity (a4) | -0.055 | | 0.073 | | -0.749 | | 0.454 | | -0.055 | | -0.053 | | |
| **Sleep during week** ~ | |  | |  | |  | |  | |  | |  |  |
| Social adversity (a5) | -0.106 | | 0.023 | | -4.590 | | 0.000 | | -0.106 | | -0.307 | | |
| **depression** ~ |  | |  | |  | |  | |  | |  | | |
| Media (b1) | 0.057 | | 0.062 | | 0.929 | | 0.353 | | 0.057 | | 0.036 | | |
| Drugs (b2) | 0.102 | | 0.095 | | 1.082 | | 0.279 | | 0.102 | | 0.053 | | |
| Alcohol (b3) | 0.019 | | 0.011 | | 1.637 | | 0.102 | | 0.019 | | 0.061 | | |
| Exercise (b4) | -0.030 | | 0.024 | | -1.249 | | 0.212 | | -0.030 | | -0.048 | | |
| Sleep during week(b5) | 0.062 | | 0.079 | | 0.788 | | 0.431 | | 0.062 | | 0.032 | | |
| **anxiety** ~ |  | |  | |  | |  | |  | |  | | |
| Media (b6) | 0.087 | | 0.056 | | 1.573 | | 0.116 | | 0.087 | | 0.068 | | |
| Drug (b7) | 0.093 | | 0.080 | | 1.158 | | 0.247 | | 0.093 | | 0.059 | | |
| Alcohol (b8) | 0.002 | | 0.011 | | 0.175 | | 0.861 | | 0.002 | | 0.007 | | |
| Exercise (b9) | -0.006 | | 0.021 | | -0.275 | | 0.784 | | -0.006 | | -0.011 | | |
| Sleep during week (b10) | -0.014 | | 0.073 | | -0.198 | | 0.843 | | -0.014 | | -0.009 | | |
| **SPQ_total** ~ |  | |  | |  | |  | |  | |  | | |
| Media (b11) | 0.247 | | 0.102 | | 2.423 | | 0.015 | | 0.247 | | 0.107 | | |
| Drug (b12) | 0.178 | | 0.124 | | 1.431 | | 0.152 | | 0.178 | | 0.063 | | |
| Alcohol (b13) | 0.011 | | 0.019 | | 0.558 | | 0.577 | | 0.011 | | 0.024 | | |
| Exercise (b14) | 0.003 | | 0.041 | | 0.061 | | 0.951 | | 0.003 | | 0.003 | | |
| Sleep during week (b15) | -0.225 | | 0.143 | | -1.576 | | 0.115 | | -0.225 | | -0.082 | | |
|  |  | |  | |  | |  | |  | |  | | |
| **Covariances:** |  | |  | |  | |  | |  | |  | | |
|  | Estimate | | Std.Err | | z-value | | P(>\|z\|) | | Std.lv | | Std.all | | |
| .anxiety ~~ |  | |  | |  | |  | |  | |  | | |
| .SPQ_total | 0.121 | | 0.022 | | 5.449 | | 0.000 | | 0.121 | | 0.433 | | |
| .depression ~~ |  | |  | |  | |  | |  | |  | | |
| .SPQ_total | 0.069 | | 0.023 | | 2.933 | | 0.003 | | 0.069 | | 0.246 | | |
| .anxiety | 0.056 | | 0.014 | | 3.870 | | 0.000 | | 0.056 | | 0.397 | | |
| livCond ~~ |  | |  | |  | |  | |  | |  | | |
| Social adversity | -0.054 | | 0.128 | | -0.424 | | 0.671 | | -0.027 | | -0.027 | | |
|  |  | |  | |  | |  | |  | |  | | |
| **Variances:** |  | |  | |  | |  | |  | |  | | |
|  | Estimate | | Std.Err | | z-value | | P(>\|z\|) | | Std.lv | | Std.all | | |
| Social adversity | 1.000 | |  | |  | |  | | 1.000 | | 1.000 | | |
| .Number rooms | 0.749 | | 0.317 | | 2.366 | | 0.018 | | 0.749 | | 0.154 | | |
| .Number people per household | 0.939 | | 0.075 | | 12.491 | | 0.000 | | 0.939 | | 0.587 | | |
| .Living Area | 1.407 | | 0.095 | | 14.871 | | 0.000 | | 1.407 | | 0.751 | | |
| .Garden_yard | 0.150 | | 0.011 | | 13.839 | | 0.000 | | 0.150 | | 0.678 | | |
| .Regular treatment physical illness | 0.078 | | 0.009 | | 8.586 | | 0.000 | | 0.078 | | 0.746 | | |
| .Mental health status before COVID19 | 0.352 | | 0.134 | | 2.627 | | 0.009 | | 0.352 | | 0.341 | | |
| .Physical health status before COVID19 | 0.592 | | 0.065 | | 9.121 | | 0.000 | | 0.592 | | 0.707 | | |
| .Lonely during COVID19 | 0.847 | | 0.088 | | 9.638 | | 0.000 | | 0.847 | | 0.596 | | |
| .Negative Thoughts during COVID19 | 0.445 | | 0.055 | | 8.154 | | 0.000 | | 0.445 | | 0.395 | | |
| .stressful social relationships changes | 0.898 | | 0.075 | | 11.918 | | 0.000 | | 0.898 | | 0.620 | | |
| .depression | 0.140 | | 0.019 | | 7.449 | | 0.000 | | 0.140 | | 0.325 | | |
| .anxiety | 0.141 | | 0.019 | | 7.359 | | 0.000 | | 0.141 | | 0.498 | | |
| .SPQ_total | 0.558 | | 0.048 | | 11.590 | | 0.000 | | 0.558 | | 0.621 | | |
| .Media | 0.150 | | 0.011 | | 13.202 | | 0.000 | | 0.150 | | 0.883 | | |
| .Drug | 0.104 | | 0.011 | | 9.929 | | 0.000 | | 0.104 | | 0.912 | | |
| .Alcohol | 4.302 | | 0.185 | | 23.263 | | 0.000 | | 4.302 | | 0.935 | | |
| .Exercise | 1.028 | | 0.073 | | 14.169 | | 0.000 | | 1.028 | | 0.962 | | |
| .Sleep during week | 0.102 | | 0.010 | | 9.704 | | 0.000 | | 0.102 | | 0.862 | | |
| Living Conditons | 4.114 | | 0.490 | | 8.396 | | 0.000 | | 1.000 | | 1.000 | | |
| .health before COVID19 | 0.023 | | 0.007 | | 3.245 | | 0.001 | | 0.883 | | 0.883 | | |
|  |  | |  | |  | |  | |  | |  | | |
| **Defined Parameters:** |  | |  | |  | |  | |  | |  | | |
|  | Estimate | | Std.Err | | z-value | | P(>\|z\|) | | Std.lv | | Std.all | | |
| indirect1 | 0.002 | | 0.003 | | 0.733 | | 0.464 | | 0.002 | | 0.004 | | |
| indirect2 | 0.005 | | 0.006 | | 0.872 | | 0.383 | | 0.005 | | 0.008 | | |
| indirect3 | -0.002 | | 0.004 | | -0.426 | | 0.670 | | -0.002 | | -0.002 | | |
| indirect4 | 0.002 | | 0.003 | | 0.566 | | 0.571 | | 0.002 | | 0.003 | | |
| indirect5 | -0.007 | | 0.009 | | -0.734 | | 0.463 | | -0.007 | | -0.010 | | |
| indirect6 | 0.004 | | 0.003 | | 1.068 | | 0.286 | | 0.004 | | 0.007 | | |
| indirect7 | 0.005 | | 0.005 | | 0.835 | | 0.404 | | 0.005 | | 0.009 | | |
| indirect8 | -0.000 | | 0.002 | | -0.080 | | 0.936 | | -0.000 | | -0.000 | | |
| indirect9 | 0.000 | | 0.002 | | 0.161 | | 0.872 | | 0.000 | | 0.001 | | |
| indirect10 | 0.002 | | 0.008 | | 0.193 | | 0.847 | | 0.002 | | 0.003 | | |
| indirect11 | 0.010 | | 0.008 | | 1.228 | | 0.219 | | 0.010 | | 0.011 | | |
| indirect12 | 0.009 | | 0.009 | | 1.008 | | 0.313 | | 0.009 | | 0.009 | | |
| indirect13 | -0.001 | | 0.004 | | -0.247 | | 0.805 | | -0.001 | | -0.001 | | |
| indirect14 | -0.000 | | 0.004 | | -0.036 | | 0.971 | | -0.000 | | -0.000 | | |
| indirect15 | 0.024 | | 0.016 | | 1.510 | | 0.131 | | 0.024 | | 0.025 | | |
| total1 | 0.466 | | 0.040 | | 11.763 | | 0.000 | | 0.466 | | 0.709 | | |
| total2 | 0.468 | | 0.042 | | 11.280 | | 0.000 | | 0.468 | | 0.713 | | |
| total3 | 0.462 | | 0.040 | | 11.591 | | 0.000 | | 0.462 | | 0.703 | | |
| total4 | 0.465 | | 0.039 | | 11.804 | | 0.000 | | 0.465 | | 0.708 | | |
| total5 | 0.457 | | 0.038 | | 12.181 | | 0.000 | | 0.457 | | 0.696 | | |
| total6 | 0.272 | | 0.040 | | 6.787 | | 0.000 | | 0.272 | | 0.510 | | |
| total7 | 0.273 | | 0.042 | | 6.480 | | 0.000 | | 0.273 | | 0.512 | | |
| total8 | 0.268 | | 0.041 | | 6.576 | | 0.000 | | 0.268 | | 0.503 | | |
| total9 | 0.268 | | 0.041 | | 6.607 | | 0.000 | | 0.268 | | 0.504 | | |
| total10 | 0.270 | | 0.040 | | 6.804 | | 0.000 | | 0.270 | | 0.506 | | |
| total11 | 0.247 | | 0.067 | | 3.666 | | 0.000 | | 0.247 | | 0.260 | | |
| total12 | 0.245 | | 0.069 | | 3.574 | | 0.000 | | 0.245 | | 0.259 | | |
| total13 | 0.236 | | 0.068 | | 3.452 | | 0.001 | | 0.236 | | 0.249 | | |
| total14 | 0.236 | | 0.068 | | 3.477 | | 0.001 | | 0.236 | | 0.249 | | |
| total15 | 0.260 | | 0.064 | | 4.099 | | 0.000 | | 0.260 | | 0.275 | | |
|  |  | |  | |  | |  | |  | |  | | |

### Social adversity Model – third timepoint

| **Suppl. file 1k. Complete outcome of structural equation with Social adversity as predictor from third survey timepoint** | | | | | | |
| --- | --- | --- | --- | --- | --- | --- |
| **Estimator** | **ML** |  |  |  |  |  |
| Optimization method | NLMINB |  |  |  |  |  |
| Number of free parameters | 108 |  |  |  |  |  |
|  | Used | Total |  |  |  |  |
| Number of observations | 488 | 532 |  |  |  |  |
| **Model Test User Model:** |  |  |  |  |  |  |
| Test statistic | 498.065 |  |  |  |  |  |
| Degrees of freedom | 135 |  |  |  |  |  |
| P-value (Chi-square) | 0.000 |  |  |  |  |  |
| **Model Test Baseline Model:** |  |  |  |  |  |  |
| Test statistic | 2937.944 |  |  |  |  |  |
| Degrees of freedom | 225 |  |  |  |  |  |
| P-value | 0.000 |  |  |  |  |  |
| **User Model versus Baseline Model:** |  |  |  |  |  |  |
| Comparative Fit Index (CFI) | 0.866 |  |  |  |  |  |
| Tucker-Lewis Index (TLI) | 0.777 |  |  |  |  |  |
| **Loglikelihood and Information Criteria:** |  |  |  |  |  |  |
| Loglikelihood user model (H0) | -10386.424 |  |  |  |  |  |
| Loglikelihood unrestricted model (H1) | -10137.391 |  |  |  |  |  |
| Akaike (AIC) | 20988.847 |  |  |  |  |  |
| Bayesian (BIC) | 21441.401 |  |  |  |  |  |
| Sample-size adjusted Bayesian (BIC) | 21098.613 |  |  |  |  |  |
| **Root Mean Square Error of Approximation:** |  |  |  |  |  |  |
| RMSEA | 0.074 |  |  |  |  |  |
| 90 Percent confidence interval - lower | 0.067 |  |  |  |  |  |
| 90 Percent confidence interval - upper | 0.081 |  |  |  |  |  |
| P-value RMSEA <= 0.05 | 0.000 |  |  |  |  |  |
| **Standardized Root Mean Square Residual:** |  |  |  |  |  |  |
| SRMR | 0.072 |  |  |  |  |  |
| **Parameter Estimates:** |  |  |  |  |  |  |
| Standard errors | Bootstrap |  |  |  |  |  |
| Number of requested bootstrap draws | 1000 |  |  |  |  |  |
| Number of successful bootstrap draws | 1000 |  |  |  |  |  |
| **Latent Variables:** |  |  |  |  |  |  |
|  | Estimate | Std.Err | z-value | P(>\|z\|) | Std.lv | Std.all |
| **Living Conditions =~** |  |  |  |  |  |  |
| Number_rooms | 1.000 |  |  |  | 1.706 | 0.798 |
| Number people per household | 0.408 | 0.041 | 9.999 | 0.000 | 0.695 | 0.527 |
| Living Area | 0.413 | 0.061 | 6.757 | 0.000 | 0.704 | 0.509 |
| Garden_yard | 0.161 | 0.022 | 7.197 | 0.000 | 0.275 | 0.599 |
| **Health before COVID19 =~** | |  |  |  |  |  |
| Regular treatment physical illness | 1.000 |  |  |  | 0.199 | 0.549 |
| Mental health status before COVID19 | -4.547 | 0.644 | -7.058 | 0.000 | -0.905 | -0.882 |
| Physical health status before COVID19 | -3.135 | 0.434 | -7.228 | 0.000 | -0.624 | -0.630 |
| **Social adversity =~** |  |  |  |  |  |  |
| Lonely during COVID19 | 0.891 | 0.056 | 15.872 | 0.000 | 0.891 | 0.698 |
| Negative Thoughts during COVID19 | 0.890 | 0.046 | 19.160 | 0.000 | 0.890 | 0.764 |
| Stressful social relationship changes | 0.879 | 0.061 | 14.331 | 0.000 | 0.879 | 0.650 |
|  |  |  |  |  |  |  |
| **Regressions:** |  |  |  |  |  |  |
|  | Estimate | Std.Err | z-value | P(>\|z\|) | Std.lv | Std.all |
| **depression** ~ |  |  |  |  |  |  |
| Health before COVID19 | -1.156 | 0.193 | -5.986 | 0.000 | -0.230 | -0.323 |
| Country (residence) | -0.186 | 0.062 | -3.020 | 0.003 | -0.186 | -0.120 |
| Age | -0.003 | 0.002 | -1.807 | 0.071 | -0.003 | -0.062 |
| Gender | 0.020 | 0.052 | 0.376 | 0.707 | 0.020 | 0.013 |
| Highest education | 0.012 | 0.015 | 0.756 | 0.449 | 0.012 | 0.024 |
| Living Conditions | 0.043 | 0.018 | 2.343 | 0.019 | 0.073 | 0.102 |
| **anxiety** ~ |  |  |  |  |  |  |
| Health before COVID19 | -0.895 | 0.197 | -4.542 | 0.000 | -0.178 | -0.322 |
| Country (residence) | -0.144 | 0.061 | -2.347 | 0.019 | -0.144 | -0.119 |
| Age | -0.005 | 0.001 | -3.539 | 0.000 | -0.005 | -0.132 |
| Gender | 0.114 | 0.041 | 2.767 | 0.006 | 0.114 | 0.094 |
| Highest education | 0.012 | 0.015 | 0.806 | 0.420 | 0.012 | 0.034 |
| Living Conditions | 0.031 | 0.016 | 1.926 | 0.054 | 0.052 | 0.094 |
| **SPQ_total** ~ |  |  |  |  |  |  |
| Health before COVID19 | -1.639 | 0.300 | -5.461 | 0.000 | -0.326 | -0.331 |
| Country (residence) | -0.089 | 0.105 | -0.851 | 0.395 | -0.089 | -0.042 |
| Age | -0.010 | 0.003 | -3.468 | 0.001 | -0.010 | -0.146 |
| Gender | 0.010 | 0.087 | 0.120 | 0.904 | 0.010 | 0.005 |
| Highest education | -0.057 | 0.028 | -2.056 | 0.040 | -0.057 | -0.086 |
| Living Conditions | -0.010 | 0.029 | -0.334 | 0.738 | -0.016 | -0.017 |
| **Media** ~ |  |  |  |  |  |  |
| Health before COVID19 | -0.017 | 0.126 | -0.138 | 0.890 | -0.003 | -0.007 |
| Country (residence) | -0.077 | 0.046 | -1.678 | 0.093 | -0.077 | -0.073 |
| Age | -0.001 | 0.001 | -0.541 | 0.588 | -0.001 | -0.024 |
| Gender | -0.143 | 0.046 | -3.080 | 0.002 | -0.143 | -0.133 |
| Highest education | -0.077 | 0.014 | -5.533 | 0.000 | -0.077 | -0.234 |
| Living Conditions | -0.041 | 0.015 | -2.692 | 0.007 | -0.070 | -0.143 |
| **Drugs** ~ |  |  |  |  |  |  |
| Health before COVID19 | -0.285 | 0.110 | -2.587 | 0.010 | -0.057 | -0.154 |
| Country (residence) | 0.051 | 0.039 | 1.305 | 0.192 | 0.051 | 0.063 |
| Age | -0.001 | 0.001 | -1.241 | 0.215 | -0.001 | -0.054 |
| Gender | 0.009 | 0.033 | 0.281 | 0.779 | 0.009 | 0.012 |
| Highest education | -0.007 | 0.011 | -0.641 | 0.522 | -0.007 | -0.028 |
| Living Conditions | -0.014 | 0.011 | -1.254 | 0.210 | -0.024 | -0.065 |
| **Alcohol** ~ |  |  |  |  |  |  |
| Health before COVID19 | 1.553 | 0.593 | 2.617 | 0.009 | 0.309 | 0.138 |
| Country (residence) | -0.226 | 0.214 | -1.059 | 0.290 | -0.226 | -0.047 |
| Age | 0.025 | 0.007 | 3.594 | 0.000 | 0.025 | 0.170 |
| Gender | -0.189 | 0.226 | -0.836 | 0.403 | -0.189 | -0.039 |
| Highest education | 0.225 | 0.069 | 3.271 | 0.001 | 0.225 | 0.151 |
| Living Conditions | -0.016 | 0.065 | -0.253 | 0.800 | -0.028 | -0.013 |
| **Exercise** ~ | |  |  |  |  |  |
| Health before COVID19 | -0.137 | 0.317 | -0.432 | 0.666 | -0.027 | -0.023 |
| Country (residence) | -0.261 | 0.117 | -2.221 | 0.026 | -0.261 | -0.104 |
| Age | 0.005 | 0.004 | 1.252 | 0.210 | 0.005 | 0.061 |
| Gender | 0.125 | 0.111 | 1.121 | 0.262 | 0.125 | 0.049 |
| Highest education | 0.155 | 0.035 | 4.466 | 0.000 | 0.155 | 0.199 |
| Living Conditions | 0.057 | 0.038 | 1.478 | 0.139 | 0.097 | 0.083 |
| **Sleep during week** ~ | |  |  |  |  |  |
| Health before COVID19 | -0.167 | 0.199 | -0.837 | 0.403 | -0.033 | -0.052 |
| Country (residence) | 0.124 | 0.071 | 1.742 | 0.082 | 0.124 | 0.090 |
| Age | -0.004 | 0.002 | -1.922 | 0.055 | -0.004 | -0.095 |
| Gender | 0.253 | 0.059 | 4.292 | 0.000 | 0.253 | 0.182 |
| Highest education | 0.025 | 0.018 | 1.380 | 0.168 | 0.025 | 0.059 |
| Living Conditions | -0.022 | 0.021 | -1.087 | 0.277 | -0.038 | -0.060 |
| **Health before COVI19** ~ | |  |  |  |  |  |
| Country (residence) | 0.051 | 0.027 | 1.930 | 0.054 | 0.258 | 0.119 |
| Age | 0.001 | 0.001 | 1.978 | 0.048 | 0.006 | 0.097 |
| Gender | -0.022 | 0.021 | -1.028 | 0.304 | -0.111 | -0.051 |
| Highest education | 0.002 | 0.007 | 0.317 | 0.751 | 0.010 | 0.016 |
| Living Conditions | 0.013 | 0.009 | 1.457 | 0.145 | 0.111 | 0.111 |
| **depression** ~ |  |  |  |  |  |  |
| Social adversity (c1) | 0.494 | 0.036 | 13.744 | 0.000 | 0.494 | 0.693 |
| **anxiet**y ~ |  |  |  |  |  |  |
| Social adversity (c2) | 0.262 | 0.034 | 7.783 | 0.000 | 0.262 | 0.473 |
| **SPQ_total** ~ |  |  |  |  |  |  |
| Social adversity (c3) | 0.234 | 0.060 | 3.886 | 0.000 | 0.234 | 0.237 |
| **Media** ~ |  |  |  |  |  |  |
| Social adversity (a1) | 0.108 | 0.025 | 4.279 | 0.000 | 0.108 | 0.222 |
| **Drugs** ~ |  |  |  |  |  |  |
| Social adversity (a2) | 0.103 | 0.022 | 4.651 | 0.000 | 0.103 | 0.280 |
| **Alcohol** ~ |  |  |  |  |  |  |
| Social adversity (a3) | 0.161 | 0.128 | 1.255 | 0.209 | 0.161 | 0.072 |
| **Exercise** ~ | |  |  |  |  |  |
| Social adversity (a4) | -0.033 | 0.062 | -0.542 | 0.587 | -0.033 | -0.029 |
| **Sleep during week** ~ | |  |  |  |  |  |
| Social adversity (a5) | -0.129 | 0.038 | -3.426 | 0.001 | -0.129 | -0.202 |
| **depression** ~ |  |  |  |  |  |  |
| Media (b1) | 0.128 | 0.054 | 2.348 | 0.019 | 0.128 | 0.087 |
| Drugs (b2) | 0.035 | 0.081 | 0.435 | 0.664 | 0.035 | 0.018 |
| Alcohol (b3) | 0.015 | 0.012 | 1.276 | 0.202 | 0.015 | 0.047 |
| Exercise (b4) | -0.037 | 0.021 | -1.757 | 0.079 | -0.037 | -0.060 |
| Sleep during week(b5) | -0.010 | 0.040 | -0.245 | 0.807 | -0.010 | -0.009 |
| **anxiety** ~ |  |  |  |  |  |  |
| Media (b6) | 0.128 | 0.051 | 2.522 | 0.012 | 0.128 | 0.113 |
| Drug (b7) | 0.080 | 0.078 | 1.021 | 0.307 | 0.080 | 0.053 |
| Alcohol (b8) | -0.011 | 0.011 | -0.986 | 0.324 | -0.011 | -0.045 |
| Exercise (b9) | 0.000 | 0.017 | 0.030 | 0.976 | 0.000 | 0.001 |
| Sleep during week (b10) | 0.022 | 0.045 | 0.496 | 0.620 | 0.022 | 0.025 |
| **SPQ_total** ~ |  |  |  |  |  |  |
| Media (b11) | 0.250 | 0.086 | 2.918 | 0.004 | 0.250 | 0.124 |
| Drug (b12) | 0.054 | 0.140 | 0.387 | 0.699 | 0.054 | 0.020 |
| Alcohol (b13) | -0.022 | 0.021 | -1.064 | 0.288 | -0.022 | -0.050 |
| Exercise (b14) | -0.003 | 0.035 | -0.083 | 0.934 | -0.003 | -0.003 |
| Sleep during week (b15) | -0.102 | 0.079 | -1.293 | 0.196 | -0.102 | -0.066 |
|  |  |  |  |  |  |  |
| **Covariances:** |  |  |  |  |  |  |
|  | Estimate | Std.Err | z-value | P(>\|z\|) | Std.lv | Std.all |
| livCond ~~ |  |  |  |  |  |  |
| Social adversity | -0.207 | 0.107 | -1.928 | 0.054 | -0.121 | -0.121 |
| .depression ~~ |  |  |  |  |  |  |
| .anxiety | 0.073 | 0.016 | 4.593 | 0.000 | 0.073 | 0.431 |
| .SPQ_total | 0.125 | 0.027 | 4.681 | 0.000 | 0.125 | 0.361 |
| .anxiety ~~ |  |  |  |  |  |  |
| .SPQ_total | 0.180 | 0.029 | 6.323 | 0.000 | 0.180 | 0.526 |
|  |  |  |  |  |  |  |
| **Variances:** |  |  |  |  |  |  |
|  | Estimate | Std.Err | z-value | P(>\|z\|) | Std.lv | Std.all |
| Social adversity | 1.000 |  |  |  | 1.000 | 1.000 |
| .Number rooms | 1.657 | 0.355 | 4.673 | 0.000 | 1.657 | 0.363 |
| .Number people per household | 1.260 | 0.160 | 7.894 | 0.000 | 1.260 | 0.723 |
| .Living Area | 1.416 | 0.111 | 12.777 | 0.000 | 1.416 | 0.741 |
| .Garden_yard | 0.135 | 0.012 | 11.172 | 0.000 | 0.135 | 0.641 |
| .Regular treatment physical illness | 0.092 | 0.009 | 9.805 | 0.000 | 0.092 | 0.699 |
| .Mental health status before COVID19 | 0.234 | 0.075 | 3.114 | 0.002 | 0.234 | 0.223 |
| .Physical health status before COVID19 | 0.592 | 0.052 | 11.425 | 0.000 | 0.592 | 0.603 |
| .Lonely during COVID19 | 0.836 | 0.071 | 11.783 | 0.000 | 0.836 | 0.513 |
| .Negative Thoughts during COVID19 | 0.566 | 0.060 | 9.358 | 0.000 | 0.566 | 0.417 |
| .stressful social relationships changes | 1.058 | 0.096 | 11.073 | 0.000 | 1.058 | 0.578 |
| .depression | 0.172 | 0.020 | 8.646 | 0.000 | 0.172 | 0.337 |
| .anxiety | 0.169 | 0.022 | 7.605 | 0.000 | 0.169 | 0.550 |
| .SPQ_total | 0.698 | 0.058 | 11.971 | 0.000 | 0.698 | 0.719 |
| .Media | 0.203 | 0.009 | 23.234 | 0.000 | 0.203 | 0.853 |
| .Drug | 0.119 | 0.009 | 12.698 | 0.000 | 0.119 | 0.878 |
| .Alcohol | 4.548 | 0.170 | 26.709 | 0.000 | 4.548 | 0.911 |
| .Exercise | 1.255 | 0.078 | 16.076 | 0.000 | 1.255 | 0.932 |
| .Sleep during week | 0.367 | 0.027 | 13.836 | 0.000 | 0.367 | 0.907 |
| Living Conditons | 2.910 | 0.431 | 6.757 | 0.000 | 1.000 | 1.000 |
| .health before COVID19 | 0.038 | 0.009 | 4.262 | 0.000 | 0.960 | 0.960 |
|  |  |  |  |  |  |  |
| **Defined Parameters:** |  |  |  |  |  |  |
|  | Estimate | Std.Err | z-value | P(>\|z\|) | Std.lv | Std.all |
| indirect1 | 0.014 | 0.006 | 2.260 | 0.024 | 0.014 | 0.019 |
| indirect2 | 0.004 | 0.008 | 0.437 | 0.662 | 0.004 | 0.005 |
| indirect3 | 0.002 | 0.003 | 0.891 | 0.373 | 0.002 | 0.003 |
| indirect4 | 0.001 | 0.003 | 0.494 | 0.621 | 0.001 | 0.002 |
| indirect5 | 0.001 | 0.005 | 0.235 | 0.814 | 0.001 | 0.002 |
| indirect6 | 0.014 | 0.006 | 2.313 | 0.021 | 0.014 | 0.025 |
| indirect7 | 0.008 | 0.008 | 1.022 | 0.307 | 0.008 | 0.015 |
| indirect8 | -0.002 | 0.003 | -0.634 | 0.526 | -0.002 | -0.003 |
| indirect9 | -0.000 | 0.001 | -0.015 | 0.988 | -0.000 | -0.000 |
| indirect10 | -0.003 | 0.006 | -0.486 | 0.627 | -0.003 | -0.005 |
| indirect11 | 0.027 | 0.011 | 2.396 | 0.017 | 0.027 | 0.028 |
| indirect12 | 0.006 | 0.014 | 0.385 | 0.700 | 0.006 | 0.006 |
| indirect13 | -0.004 | 0.005 | -0.711 | 0.477 | -0.004 | -0.004 |
| indirect14 | 0.000 | 0.002 | 0.041 | 0.968 | 0.000 | 0.000 |
| indirect15 | 0.013 | 0.011 | 1.219 | 0.223 | 0.013 | 0.013 |
| total1 | 0.508 | 0.036 | 14.194 | 0.000 | 0.508 | 0.713 |
| total2 | 0.498 | 0.035 | 14.254 | 0.000 | 0.498 | 0.698 |
| total3 | 0.497 | 0.036 | 13.776 | 0.000 | 0.497 | 0.697 |
| total4 | 0.496 | 0.036 | 13.738 | 0.000 | 0.496 | 0.695 |
| total5 | 0.496 | 0.035 | 14.081 | 0.000 | 0.496 | 0.695 |
| total6 | 0.276 | 0.033 | 8.301 | 0.000 | 0.276 | 0.498 |
| total7 | 0.270 | 0.035 | 7.790 | 0.000 | 0.270 | 0.487 |
| total8 | 0.260 | 0.033 | 7.781 | 0.000 | 0.260 | 0.469 |
| total9 | 0.262 | 0.034 | 7.793 | 0.000 | 0.262 | 0.473 |
| total10 | 0.259 | 0.032 | 8.022 | 0.000 | 0.259 | 0.467 |
| total11 | 0.261 | 0.059 | 4.404 | 0.000 | 0.261 | 0.265 |
| total12 | 0.239 | 0.058 | 4.151 | 0.000 | 0.239 | 0.243 |
| total13 | 0.230 | 0.060 | 3.845 | 0.000 | 0.230 | 0.234 |
| total14 | 0.234 | 0.060 | 3.893 | 0.000 | 0.234 | 0.237 |
| total15 | 0.247 | 0.058 | 4.252 | 0.000 | 0.247 | 0.250 |
|  |  |  |  |  |  |  |

### Social adversity Model – fourth timepoint

| **Suppl. file 1l. Complete outcome of structural equation with Social adversity as predictor from fourth survey timepoint** | | | | | | |
| --- | --- | --- | --- | --- | --- | --- |
| **Estimator** | **ML** |  |  |  |  |  |
| Optimization method | NLMINB |  |  |  |  |  |
| Number of free parameters | 108 |  |  |  |  |  |
|  | Used | Total |  |  |  |  |
| Number of observations | 440 | 478 |  |  |  |  |
| **Model Test User Model:** |  |  |  |  |  |  |
| Test statistic | 448.081 |  |  |  |  |  |
| Degrees of freedom | 135 |  |  |  |  |  |
| P-value (Chi-square) | 0.000 |  |  |  |  |  |
| **Model Test Baseline Model:** |  |  |  |  |  |  |
| Test statistic | 2567.853 |  |  |  |  |  |
| Degrees of freedom | 225 |  |  |  |  |  |
| P-value | 0.000 |  |  |  |  |  |
| **User Model versus Baseline Model:** |  |  |  |  |  |  |
| Comparative Fit Index (CFI) | 0.866 |  |  |  |  |  |
| Tucker-Lewis Index (TLI) | 0.777 |  |  |  |  |  |
| **Loglikelihood and Information Criteria:** |  |  |  |  |  |  |
| Loglikelihood user model (H0) | -9024.252 |  |  |  |  |  |
| Loglikelihood unrestricted model (H1) | -8800.211 |  |  |  |  |  |
| Akaike (AIC) | 18264.504 |  |  |  |  |  |
| Bayesian (BIC) | 18705.875 |  |  |  |  |  |
| Sample-size adjusted Bayesian (BIC) | 18363.135 |  |  |  |  |  |
| **Root Mean Square Error of Approximation:** | |  |  |  |  |  |
| RMSEA | 0.073 |  |  |  |  |  |
| 90 Percent confidence interval - lower | 0.065 |  |  |  |  |  |
| 90 Percent confidence interval - upper | 0.080 |  |  |  |  |  |
| P-value RMSEA <= 0.05 | 0.000 |  |  |  |  |  |
| **Standardized Root Mean Square Residual:** | | |  |  |  |  |
| SRMR | 0.070 |  |  |  |  |  |
| **Parameter Estimates:** |  |  |  |  |  |  |
| Standard errors | Bootstrap |  |  |  |  |  |
| Number of requested bootstrap draws | 1000 |  |  |  |  |  |
| Number of successful bootstrap draws | 1000 |  |  |  |  |  |
| **Latent Variables:** |  |  |  |  |  |  |
|  | Estimate | Std.Err | z-value | P(>\|z\|) | Std.lv | Std.all |
| **Living Conditions =~** |  |  |  |  | 1.786 | 0.853 |
| Number_rooms | 1.000 |  |  |  |  |  |
| Number people per household | 0.389 | 0.035 | 11.025 | 0.000 | 0.694 | 0.592 |
| Living Area | 0.345 | 0.052 | 6.693 | 0.000 | 0.616 | 0.459 |
| Garden_yard | 0.164 | 0.020 | 8.248 | 0.000 | 0.293 | 0.618 |
| **Health before COVID19 =~** | |  |  |  |  |  |
| Regular treatment physical illness | 1.000 |  |  |  | 0.148 | 0.442 |
| Mental health status before COVID19 | -5.515 | 1.265 | -4.359 | 0.000 | -0.819 | -0.808 |
| Physical health status before COVID19 | -4.094 | 0.923 | -4.435 | 0.000 | -0.608 | -0.656 |
| **Social adversity =~** |  |  |  |  |  |  |
| Lonely during COVID19 | 0.888 | 0.059 | 14.941 | 0.000 | 0.888 | 0.682 |
| Negative Thoughts during COVID19 | 0.943 | 0.044 | 21.353 | 0.000 | 0.943 | 0.826 |
| Stressful social relationship changes | 0.744 | 0.063 | 11.904 | 0.000 | 0.744 | 0.588 |
|  |  |  |  |  |  |  |
| **Regressions:** |  |  |  |  |  |  |
|  | Estimate | Std.Err | z-value | P(>\|z\|) | Std.lv | Std.all |
| **depression** ~ | |  |  |  |  |  |
| Health before COVID19 | -1.419 | 0.333 | -4.267 | 0.000 | -0.211 | -0.285 |
| Country (residence) | -0.098 | 0.067 | -1.472 | 0.141 | -0.098 | -0.055 |
| Age | -0.002 | 0.002 | -1.139 | 0.255 | -0.002 | -0.035 |
| Gender | -0.103 | 0.059 | -1.760 | 0.078 | -0.103 | -0.060 |
| Highest education | -0.060 | 0.020 | -3.006 | 0.003 | -0.060 | -0.113 |
| Living Conditions | 0.003 | 0.019 | 0.160 | 0.873 | 0.005 | 0.007 |
| **anxiety** ~ |  |  |  |  |  |  |
| Health before COVID19 | -1.245 | 0.263 | -4.741 | 0.000 | -0.185 | -0.345 |
| Country (residence) | -0.179 | 0.064 | -2.798 | 0.005 | -0.179 | -0.139 |
| Age | -0.005 | 0.001 | -3.849 | 0.000 | -0.005 | -0.148 |
| Gender | -0.049 | 0.054 | -0.907 | 0.364 | -0.049 | -0.039 |
| Highest education | -0.062 | 0.016 | -3.790 | 0.000 | -0.062 | -0.162 |
| Living Conditions | -0.007 | 0.015 | -0.481 | 0.630 | -0.013 | -0.024 |
| **SPQ_total** ~ | |  |  |  |  |  |
| Health before COVID19 | -2.342 | 0.554 | -4.225 | 0.000 | -0.348 | -0.358 |
| Country (residence) | -0.139 | 0.131 | -1.062 | 0.288 | -0.139 | -0.060 |
| Age | -0.006 | 0.003 | -2.025 | 0.043 | -0.006 | -0.089 |
| Gender | -0.261 | 0.101 | -2.590 | 0.010 | -0.261 | -0.115 |
| Highest education | -0.135 | 0.033 | -4.115 | 0.000 | -0.135 | -0.194 |
| Living Conditions | -0.039 | 0.026 | -1.481 | 0.139 | -0.070 | -0.072 |
| **Media** ~ | |  |  |  |  |  |
| Health before COVID19 | -0.303 | 0.192 | -1.583 | 0.113 | -0.045 | -0.097 |
| Country (residence) | -0.068 | 0.052 | -1.309 | 0.191 | -0.068 | -0.061 |
| Age | -0.000 | 0.002 | -0.135 | 0.893 | -0.000 | -0.007 |
| Gender | -0.174 | 0.051 | -3.382 | 0.001 | -0.174 | -0.160 |
| Highest education | -0.039 | 0.016 | -2.369 | 0.018 | -0.039 | -0.116 |
| Living Conditions | -0.024 | 0.014 | -1.639 | 0.101 | -0.042 | -0.091 |
| **Drugs** ~ | |  |  |  |  |  |
| Health before COVID19 | -0.489 | 0.157 | -3.120 | 0.002 | -0.073 | -0.194 |
| Country (residence) | -0.008 | 0.045 | -0.179 | 0.858 | -0.008 | -0.009 |
| Age | 0.002 | 0.001 | 1.468 | 0.142 | 0.002 | 0.067 |
| Gender | -0.015 | 0.042 | -0.353 | 0.724 | -0.015 | -0.017 |
| Highest education | -0.040 | 0.013 | -3.036 | 0.002 | -0.040 | -0.150 |
| Living Conditions | -0.024 | 0.011 | -2.207 | 0.027 | -0.042 | -0.113 |
| **Alcohol** ~ | |  |  |  |  |  |
| Health before COVID19 | 2.759 | 1.138 | 2.425 | 0.015 | 0.410 | 0.182 |
| Country (residence) | 0.056 | 0.266 | 0.210 | 0.834 | 0.056 | 0.010 |
| Age | 0.023 | 0.008 | 2.958 | 0.003 | 0.023 | 0.149 |
| Gender | -0.458 | 0.246 | -1.864 | 0.062 | -0.458 | -0.087 |
| Highest education | 0.218 | 0.080 | 2.728 | 0.006 | 0.218 | 0.135 |
| Living Conditions | 0.077 | 0.066 | 1.168 | 0.243 | 0.138 | 0.061 |
| **Exercise** ~ | |  |  |  |  |  |
| Health before COVID19 | 0.182 | 0.481 | 0.379 | 0.705 | 0.027 | 0.024 |
| Country (residence) | -0.053 | 0.145 | -0.363 | 0.716 | -0.053 | -0.020 |
| Age | 0.002 | 0.004 | 0.486 | 0.627 | 0.002 | 0.026 |
| Gender | -0.050 | 0.134 | -0.374 | 0.708 | -0.050 | -0.019 |
| Highest education | 0.140 | 0.036 | 3.887 | 0.000 | 0.140 | 0.175 |
| Living Conditions | 0.030 | 0.039 | 0.766 | 0.444 | 0.054 | 0.049 |
| **Sleep during week** ~ | |  |  |  |  |  |
| Health before COVID19 | 0.190 | 0.172 | 1.107 | 0.268 | 0.028 | 0.070 |
| Country (residence) | 0.053 | 0.051 | 1.037 | 0.300 | 0.053 | 0.055 |
| Age | -0.001 | 0.001 | -0.600 | 0.549 | -0.001 | -0.026 |
| Gender | 0.066 | 0.046 | 1.441 | 0.150 | 0.066 | 0.071 |
| Highest education | 0.038 | 0.014 | 2.687 | 0.007 | 0.038 | 0.131 |
| Living Conditions | 0.004 | 0.012 | 0.357 | 0.721 | 0.007 | 0.018 |
| **Health before COVI19** ~ | |  |  |  |  |  |
| Country (residence) | 0.046 | 0.022 | 2.062 | 0.039 | 0.308 | 0.128 |
| Age | 0.001 | 0.001 | 1.487 | 0.137 | 0.006 | 0.091 |
| Gender | 0.008 | 0.019 | 0.429 | 0.668 | 0.054 | 0.023 |
| Highest education | -0.009 | 0.006 | -1.455 | 0.146 | -0.059 | -0.082 |
| Living Conditions | -0.001 | 0.006 | -0.195 | 0.846 | -0.014 | -0.014 |
| **depression** ~ | |  |  |  |  |  |
| Social adversity (c1) | 0.552 | 0.035 | 15.699 | 0.000 | 0.552 | 0.747 |
| **anxiet**y ~ |  |  |  |  |  |  |
| Social adversity (c2) | 0.287 | 0.030 | 9.465 | 0.000 | 0.287 | 0.535 |
| **SPQ_total** ~ | |  |  |  |  |  |
| Social adversity (c3) | 0.328 | 0.062 | 5.315 | 0.000 | 0.328 | 0.337 |
| **Media** ~ | |  |  |  |  |  |
| Social adversity (a1) | 0.051 | 0.027 | 1.915 | 0.056 | 0.051 | 0.111 |
| **Drugs** ~ | |  |  |  |  |  |
| Social adversity (a2) | 0.058 | 0.024 | 2.386 | 0.017 | 0.058 | 0.154 |
| **Alcohol** ~ | |  |  |  |  |  |
| Social adversity (a3) | 0.053 | 0.135 | 0.395 | 0.693 | 0.053 | 0.024 |
| **Exercise** ~ | |  |  |  |  |  |
| Social adversity (a4) | -0.048 | 0.061 | -0.782 | 0.434 | -0.048 | -0.043 |
| **Sleep during week** ~ | |  |  |  |  |  |
| Social adversity (a5) | -0.095 | 0.024 | -4.048 | 0.000 | -0.095 | -0.238 |
| **depression** ~ | |  |  |  |  |  |
| Media (b1) | 0.058 | 0.060 | 0.969 | 0.333 | 0.058 | 0.037 |
| Drugs (b2) | 0.088 | 0.083 | 1.064 | 0.287 | 0.088 | 0.045 |
| Alcohol (b3) | 0.019 | 0.013 | 1.443 | 0.149 | 0.019 | 0.058 |
| Exercise (b4) | -0.006 | 0.024 | -0.260 | 0.795 | -0.006 | -0.009 |
| Sleep during week(b5) | 0.013 | 0.075 | 0.180 | 0.857 | 0.013 | 0.007 |
| **anxiety** ~ |  |  |  |  |  |  |
| Media (b6) | 0.087 | 0.046 | 1.894 | 0.058 | 0.087 | 0.076 |
| Drug (b7) | -0.021 | 0.075 | -0.283 | 0.777 | -0.021 | -0.015 |
| Alcohol (b8) | 0.007 | 0.010 | 0.658 | 0.511 | 0.007 | 0.028 |
| Exercise (b9) | 0.049 | 0.019 | 2.542 | 0.011 | 0.049 | 0.102 |
| Sleep during week (b10) | 0.033 | 0.062 | 0.529 | 0.597 | 0.033 | 0.025 |
| **SPQ_total** ~ | |  |  |  |  |  |
| Media (b11) | -0.001 | 0.092 | -0.016 | 0.987 | -0.001 | -0.001 |
| Drug (b12) | 0.113 | 0.135 | 0.836 | 0.403 | 0.113 | 0.044 |
| Alcohol (b13) | -0.017 | 0.020 | -0.869 | 0.385 | -0.017 | -0.040 |
| Exercise (b14) | 0.045 | 0.042 | 1.083 | 0.279 | 0.045 | 0.052 |
| Sleep during week (b15) | 0.051 | 0.140 | 0.363 | 0.717 | 0.051 | 0.021 |
|  |  |  |  |  |  |  |
| **Covariances:** |  |  |  |  |  |  |
|  | Estimate | Std.Err | z-value | P(>\|z\|) | Std.lv | Std.all |
| .anxiety ~~ |  |  |  |  |  |  |
| .SPQ_total | 0.132 | 0.024 | 5.514 | 0.000 | 0.132 | 0.438 |
| .depression ~~ |  |  |  |  |  |  |
| .SPQ_total | 0.073 | 0.024 | 2.967 | 0.003 | 0.073 | 0.220 |
| .anxiety | 0.048 | 0.014 | 3.354 | 0.001 | 0.048 | 0.302 |
| livCond ~~ |  |  |  |  |  |  |
| Social adversity | -0.251 | 0.119 | -2.109 | 0.035 | -0.141 | -0.141 |
|  |  |  |  |  |  |  |
| **Variances:** |  |  |  |  |  |  |
|  | Estimate | Std.Err | z-value | P(>\|z\|) | Std.lv | Std.all |
| Social adversity | 1.000 |  |  |  | 1.000 | 1.000 |
| .Number rooms | 1.196 | 0.312 | 3.833 | 0.000 | 1.196 | 0.273 |
| .Number people per household | 0.894 | 0.063 | 14.231 | 0.000 | 0.894 | 0.650 |
| .Living Area | 1.426 | 0.102 | 13.971 | 0.000 | 1.426 | 0.790 |
| .Garden_yard | 0.139 | 0.012 | 11.508 | 0.000 | 0.139 | 0.618 |
| .Regular treatment physical illness | 0.091 | 0.009 | 9.569 | 0.000 | 0.091 | 0.805 |
| .Mental health status before COVID19 | 0.357 | 0.098 | 3.624 | 0.000 | 0.357 | 0.347 |
| .Physical health status before COVID19 | 0.489 | 0.060 | 8.097 | 0.000 | 0.489 | 0.570 |
| .Lonely during COVID19 | 0.908 | 0.088 | 10.365 | 0.000 | 0.908 | 0.535 |
| .Negative Thoughts during COVID19 | 0.415 | 0.054 | 7.623 | 0.000 | 0.415 | 0.318 |
| .stressful social relationships changes | 1.047 | 0.085 | 12.263 | 0.000 | 1.047 | 0.654 |
| .depression | 0.173 | 0.022 | 7.971 | 0.000 | 0.173 | 0.317 |
| .anxiety | 0.144 | 0.017 | 8.434 | 0.000 | 0.144 | 0.503 |
| .SPQ_total | 0.632 | 0.062 | 10.163 | 0.000 | 0.632 | 0.670 |
| .Media | 0.199 | 0.009 | 21.337 | 0.000 | 0.199 | 0.927 |
| .Drug | 0.126 | 0.011 | 11.602 | 0.000 | 0.126 | 0.903 |
| .Alcohol | 4.635 | 0.202 | 22.916 | 0.000 | 4.635 | 0.911 |
| .Exercise | 1.185 | 0.084 | 14.115 | 0.000 | 1.185 | 0.962 |
| .Sleep during week | 0.147 | 0.010 | 14.163 | 0.000 | 0.147 | 0.915 |
| Living Conditons | 3.189 | 0.415 | 7.683 | 0.000 | 1.000 | 1.000 |
| .health before COVID19 | 0.021 | 0.008 | 2.745 | 0.006 | 0.966 | 0.966 |
|  |  |  |  |  |  |  |
| **Defined Parameters:** |  |  |  |  |  |  |
|  | Estimate | Std.Err | z-value | P(>\|z\|) | Std.lv | Std.all |
| indirect1 | 0.003 | 0.003 | 0.855 | 0.393 | 0.003 | 0.004 |
| indirect2 | 0.005 | 0.006 | 0.911 | 0.362 | 0.005 | 0.007 |
| indirect3 | 0.001 | 0.003 | 0.322 | 0.747 | 0.001 | 0.001 |
| indirect4 | 0.000 | 0.002 | 0.156 | 0.876 | 0.000 | 0.000 |
| indirect5 | -0.001 | 0.008 | -0.166 | 0.868 | -0.001 | -0.002 |
| indirect6 | 0.004 | 0.003 | 1.305 | 0.192 | 0.004 | 0.008 |
| indirect7 | -0.001 | 0.005 | -0.260 | 0.795 | -0.001 | -0.002 |
| indirect8 | 0.000 | 0.002 | 0.200 | 0.842 | 0.000 | 0.001 |
| indirect9 | -0.002 | 0.003 | -0.699 | 0.485 | -0.002 | -0.004 |
| indirect10 | -0.003 | 0.006 | -0.492 | 0.623 | -0.003 | -0.006 |
| indirect11 | -0.000 | 0.006 | -0.013 | 0.989 | -0.000 | -0.000 |
| indirect12 | 0.007 | 0.009 | 0.746 | 0.455 | 0.007 | 0.007 |
| indirect13 | -0.001 | 0.004 | -0.240 | 0.810 | -0.001 | -0.001 |
| indirect14 | -0.002 | 0.004 | -0.515 | 0.606 | -0.002 | -0.002 |
| indirect15 | -0.005 | 0.014 | -0.337 | 0.736 | -0.005 | -0.005 |
| total1 | 0.555 | 0.035 | 15.789 | 0.000 | 0.555 | 0.751 |
| total2 | 0.557 | 0.035 | 15.756 | 0.000 | 0.557 | 0.754 |
| total3 | 0.553 | 0.035 | 15.671 | 0.000 | 0.553 | 0.748 |
| total4 | 0.552 | 0.035 | 15.732 | 0.000 | 0.552 | 0.747 |
| total5 | 0.550 | 0.034 | 15.997 | 0.000 | 0.550 | 0.745 |
| total6 | 0.291 | 0.030 | 9.581 | 0.000 | 0.291 | 0.544 |
| total7 | 0.285 | 0.030 | 9.452 | 0.000 | 0.285 | 0.533 |
| total8 | 0.287 | 0.030 | 9.515 | 0.000 | 0.287 | 0.536 |
| total9 | 0.284 | 0.030 | 9.353 | 0.000 | 0.284 | 0.531 |
| total10 | 0.283 | 0.029 | 9.849 | 0.000 | 0.283 | 0.530 |
| total11 | 0.328 | 0.061 | 5.335 | 0.000 | 0.328 | 0.337 |
| total12 | 0.334 | 0.062 | 5.422 | 0.000 | 0.334 | 0.344 |
| total13 | 0.327 | 0.061 | 5.334 | 0.000 | 0.327 | 0.336 |
| total14 | 0.325 | 0.062 | 5.277 | 0.000 | 0.325 | 0.335 |
| total15 | 0.323 | 0.056 | 5.801 | 0.000 | 0.323 | 0.332 |
|  |  |  |  |  |  |  |

# Model with reduced in complexity, with one predictor and one outcome without control variables

| **Suppl. file 1m. Model fit for model with reduced complexity, one predictor, one outcome, five mediators, example for timepoint 1** | | | | | | |
| --- | --- | --- | --- | --- | --- | --- |
|  | Fit index: | Teststatistic | DF | *X^2^* | CFI | RMSEA |
| Predictor | Outcome |  |  |  |  |  |
| COVID-19 related life concerns | SPQ | 40.31 | 22 | .010 | 0.922 | 0.042 |
|  | Anxiety | 61.28 | 22 | .000 | 0.843 | 0.061 |
|  | Depression | 83.31 | 22 | .000 | 0.817 | 0.076 |
| Social adversity | SPQ | 46.51 | 22 | .002 | 0.938 | 0.048 |
|  | Anxiety | 43.13 | 22 | .005 | 0.955 | 0.045 |
|  | Depression | 46.71 | 22 | .002 | 0.962 | 0.048 |
| DF: degrees of freedom, *X^2^:* Chi squared, CFI: Comparative Fit Index; RMSEA: Root Mean Square Error of Approximation | | | | | | |

# Alternative models

## COVID-19 related life concerns’ model – alternative models

The alternative models for the different samples at the four different time points evaluating the effect of ‘COVID-19 related life concerns’ -> schizotypy/anxiety/depression -> alcohol/media/drugs/sleep/exercise revealed worse fit than our first proposed model of of ‘COVID-19 related life concerns’ -> alcohol/media/drugs/sleep/exercise -> schizotyoy/anxiety/depression. See **Suppl.** **Table 7,** for AIC/BIC in comparison.

## ‘Social adversity’ Model – alternative models

The alternative models evaluating the effect of ‘social adversity -> schizotypy/anxiety/depression -> alcohol/media/drugs/sleep/exercise revealed worse fit than our first proposed model of of ‘social adversity’ -> alcohol/media/drugs/sleep/exercise -> schizotypy/anxiety/depression. See **Suppl.** **Table 7** for AIC/BIC in comparison.

| **Suppl. file 1n. Model comparison for original and alternative model.** | | | | | | | | |
| --- | --- | --- | --- | --- | --- | --- | --- | --- |
|  | Co19 life concerns | | alternative model | | Social adversity | | alternative model | |
| Timepoint | AIC | BIC | AIC | BIC | AIC | BIC | AIC | BIC |
| 1 | 20691.60 | 21142.37 | 20753.43 | 21233.42 | 20394.58 | 20845.35 | 20443.93 | 20923.92 |
| 2 | 17023.27 | 17462.41 | 17082.42 | 17550.02 | 16811.99 | 17249.11 | 16865.25 | 17330.70 |
| 3 | 21325.87 | 21780.18 | 21464.28 | 21948.03 | 20988.85 | 21441.40 | 21109.03 | 21590.92 |
| 4 | 18711.20 | 19155.48 | 18791.80 | 19264.87 | 18264.50 | 18705.88 | 18326.27 | 18796.25 |
| AIC: Akaike information criterion, BIC: Bayesian information criterion | | | | | | | | |

# **Exploratory model COVID-stress -> Anxiety/Depression -> SPQ**

| **Suppl. file 1o. Overview of the model fit indices separated by exogeneous latent variable and time point** | | | | | | | | | | |
| --- | --- | --- | --- | --- | --- | --- | --- | --- | --- | --- |
|  |  |  |  | exact modelfit |  | relativ modelfit | |  | absolute modelfit | |
|  |  | Teststatistic | DF | *X^2^* |  | CFI | TLI |  | RMSEA | |
| Predictor | Timepoint |  |  |  |  |  |  |  |  | |
| COVID-19 related life concerns | 1 | 396.76 | 90 | .000 |  | 0.838 | 0.765 |  | 0.084 | |
|  | 2 | 359.18 | 90 | .000 |  | 0.866 | 0.807 |  | 0.083 | |
|  | 3 | 359.08 | 90 | .000 |  | 0.880 | 0.827 |  | 0.077 | |
|  | 4 | 332.55 | 90 | .000 |  | 0.871 | 0.814 |  | 0.077 | |
| Social adversity | 1 | 416.14 | 90 | .000 |  | 0.853 | 0.787 |  | 0.087 | |
|  | 2 | 413.23 | 90 | .000 |  | 0.854 | 0.790 |  | 0.092 | |
|  | 3 | 411.72 | 90 | .000 |  | 0.868 | 0.809 |  | 0.085 | |
|  | 4 | 347.08 | 90 | .000 |  | 0.880 | 0.826 |  | 0.081 | |
| DF: degree of freedom, *X^2^*: Chi squared test, CFI: comparative fit index, TLI: Tucker-Lewis index, RMSEA: root mean square error of approximation | | | | | | | | | |  |
